# Spatial and temporal distribution of the prevalence of unemployment and early retirement in people with multiple sclerosis: A systematic review with meta-analysis

**DOI:** 10.1371/journal.pone.0272156

**Published:** 2022-07-28

**Authors:** Bruno Kusznir Vitturi, Alborz Rahmani, Guglielmo Dini, Alfredo Montecucco, Nicoletta Debarbieri, Paolo Bandiera, Mario Alberto Battaglia, Tommaso Manacorda, Benedetta Persechino, Giuliana Buresti, Michela Ponzio, Matilde Inglese, Paolo Durando

**Affiliations:** 1 Department of Health Sciences, University of Genoa, Genoa, Italy; 2 IRCCS Ospedale Policlinico San Martino, Occupational Medicine Unit, Genoa, Italy; 3 Italian Multiple Sclerosis Association (AISM), Genoa, Italy; 4 Scientific Research Area, Italian Multiple Sclerosis Foundation (FISM), Genoa, Italy; 5 Department of Life Science, University of Siena, Siena, Italy; 6 Italian Workers’ Compensation Authority (INAIL), Genoa, Italy; 7 Department of Neurosciences, Rehabilitation, Ophthalmology, Genetics, Maternal and Child Health (DiNOGMI) and Center of Excellence for Biomedical Research (CEBR), University of Genoa, Genoa, Italy; 8 IRCCS Ospedale Policlinico San Martino, Genoa, Italy; Universita degli Studi di Napoli Federico II, ITALY

## Abstract

**Background:**

We aimed to summarise the prevalence of unemployment and early retirement among people with MS and analyze data according to a spatio-temporal perspective.

**Methods:**

We undertook a systematic search of PubMed/MEDLINE, Scopus, SciVerse ScienceDirect, and Web of Science. We included any peer-reviewed original article reporting the prevalence of unemployment and early retirement in the working-age population with MS. We excluded articles off-topic, with other study designs, whose study sample were unlikely to be representative of the MS population and in case of unavailability of the full text or essential information. A random-effects meta-analysis was used to measure overall prevalence estimates of unemployment and early retirement. We used meta-regression and subgroup analysis to evaluate potential moderators of prevalence estimates and the leave-one-out method for sensitivity analyses.

**Results:**

Our research identified 153 studies across 29 countries encompassing 188436 subjects with MS. The pooled overall effect size for unemployment and early retirement was 35.6% (95% CI 32.8–38.4; I^2^ = 99.31) and 17.2% (95% CI 14.6–20.2; I^2^ = 99.13), respectively. The prevalence of unemployment varied according to the year of publication (p < 0.001) and there was a statistically significant decrease in the prevalence of unemployment over time (p = 0.042). Regarding early retirement, only seven (31.8%) estimates obtained from studies that were published before 2010 were below the overall effect size in comparison to 27 (60.0%) estimates extracted from data published between 2010 and 2021 (p = 0.039). There was a significant difference in prevalence according to countries (p < 0.001). Psychiatric illness was an important clinical feature responsible for patients leaving the workforce in regions with a high MS prevalence.

**Conclusions:**

Unemployment and early retirement due to MS remain highly prevalent, despite a slight decline in the last decade. The prevalence of unemployment and early retirement varies globally.

## Introduction

Multiple Sclerosis (MS) is a chronic autoimmune disease that causes demyelination and neurodegeneration in the central nervous system. It mainly affects young people between 20 and 40 years of age and it is the main cause of non-traumatic disability among young adults in the Western world [1]. About 2.8 million people worldwide suffer from MS, whose incidence and prevalence increase in both developed and developing countries [2]. The symptoms are extremely varied and the clinical course is within a spectrum that extends from relapsing-remitting to progressive [3].

Besides the inherent clinical complexity of MS, the age of onset of the disease brings inevitable repercussions to work activity, once it coincides with the moment in which people with MS (PwMS) find themselves managing the already expected difficulties of the job market and the beginning of the professional career [4, 5]. Often limiting and disabling, symptoms such as fatigue, neuropsychiatric impairment, and motor disturbances constantly threaten the full performance at work and the search for new professional skills [6, 7]. PwMS are vulnerable to barriers related to the work environment (e.g. high temperature level, difficult access to the workplace, noise) deterioration of social relationships at work, negative work events and stigma and discrimination in the workplace. Moreover, intrinsic characteristics of the job such as inflexible work schedules and extended standing time can make work unviable for PwMS [8–10].

MS is recognized as a well-known risk factor for unemployment and early retirement. A Norwegian study found that after 19 years of disease, only 45% of patients were still employed [11]. In a Swedish cohort, only 28% and 23% of PwMS were working full- and part-time after a follow-up of ten years, respectively [12]. In 2013, Krause et al. showed that 44.8% of PwMS were forced to retire early due to their illness [13]. Once unemployed, PwMS face substantial difficulties to return to the workforce [14]. In addition to the undeniable importance that work plays in people’s lives and the financial and psychological consequences that the loss of a job can entail, unemployed and early retired patients are known to be associated with a worse level of quality of life [15].

Although unemployment and early retirement are already sufficiently eloquent consequences in the personal life of PwMS, it is impossible not to recognize the economic burden closely associated with these two outcomes. In Germany, approximately 27.300 persons received early retirement pensions caused by MS [16]. Battaglia et al. showed that invalidity and early retirement can cost more than 18.000 € per patient every year [17]. Indeed, it is not frivolous to affirm that MS is one of the most costly diseases, once it dialogues with the global economy and the public health closely [18].

If, on the one hand, the literature is relatively abundant concerning data on unemployment and early retirement in MS, on the other hand, there is an enormous diversity of data that prevents clinicians and researchers from having the real dimension of this issue. In fact, there is no study aimed at systematically synthesising the available data. Over time, there have been remarkable advances in the understanding of MS and its treatment and, since 2010, several disease-modifying drugs (DMDs) have been approved [19]. Nevertheless, there is still no evidence indicating the temporal evolution of the occupational outcomes in PwMS. Moreover, there is also a complete lack of studies describing and comparing the prevalence of unemployment and early retirement considering a geographical point of view. Strategies to prevent these outcomes are complex and can vary substantially across countries. An accurate understanding of the geographical particularities of unemployment and early retirement is crucial to guide effective strategies to promote the integration of PwMS into work.

The influence of MS on unemployment and early retirement is a public health issue. In almost 40 years of published data on the prevalence of unemployment and early retirement in workers with MS, it is imperative to understand the full epidemiological and occupational context of the disease. Effective public health strategies depend on this type of approach and are crucial to promote the occupational outcomes and the quality of life of PwMS. Aware of this scenario and the importance of this topic, we performed the first systematic review with meta-analysis that address the prevalence of unemployment and early retirement in a temporal-spatial perspective. The review aimed to summarize the prevalence of unemployment and early retirement among PwMS, describe if there has been any significant change over time, and compare these two outcomes from a geographical point of view.

## Materials and methods

This study was carried out according to the Preferred Reporting Items for Systematic Reviews and Meta-Analyses Statement (PRISMA) [20] (S1 File) and the Joanna Briggs recommendations for systematic reviews of observational epidemiological studies reporting prevalence and cumulative incidence data [21]. The protocol was registered in PROSPERO (CRD42021285216). As this was a literature review, ethical approval wasn’t necessary as it didn’t involve the recruitment of subjects and data were analyzed from already published original articles.

### Search strategy and selection criteria

From August 1, 2021, to October 30, 2021, we systematically searched on PubMed/MEDLINE, Scopus, SciVerse ScienceDirect, and Web of Science the following keywords (Employ* OR unemploy* OR occupation* OR “work” OR vocation* OR “work resumption” OR workplace* OR “return to work” OR “workforce” OR “workforce” OR “labour force” OR “labor force” OR Career* OR Job* OR “job retention” OR retire* OR “disability pension” OR “worker” OR “fitness for work”) AND (“Multiple sclerosis” OR “Disseminated Sclerosis” OR “Demyelinating Autoimmune Diseases” OR “Demyelinating Autoimmune Disorders” OR “Clinically Isolated Syndrome” OR “Demyelinating”). The details of the search strategy used are reported in S1 Fig. We didn’t explore any grey literature sources. We adopted a broad search methodology to ensure the maximum inclusion of studies reporting both outcomes.

Articles were selected according to the CoCoPop (Condition/Context/Population) strategy. We included any peer-reviewed original article reporting the prevalence of unemployment and early retirement of PwMS in the working age. MS must have been diagnosed according to accepted international criteria at the time of the study and/or confirmed by a doctor. No time limits were set for the search. We included articles whose full text was published in English, Italian, Spanish, French, and Portuguese. As this is a systematic review of epidemiological articles, studies should contain a minimum sample of 124 subjects. This number was calculated according to the formula n = [Z^2^. P(1—P)] / d^2^, where *n* is the sample size, *Z* is the Z statistic for a given level of confidence (1.96), *P* is the expected global prevalence, and *d* is the precision (in a proportion of one; if 5%, d = 0.05). Data were taken from cross-sectional cohort studies and baseline measurements in longitudinal and interventional studies with clinical follow-up.

After we removed duplicate entries, we performed an initial screen of titles or abstracts to assess potential relevance and remove those off-topic. Screening of titles, abstracts, and full texts for each article was conducted by three experienced and trained investigators (BKV, AR, and AM), each blinded to the other’s ratings. In case of discrepancy, a final decision was made by a consensus. Afterward, we obtained relevant full-text articles, revaluated their eligibility, and determined their final inclusion or exclusion.

**Fig 1:**
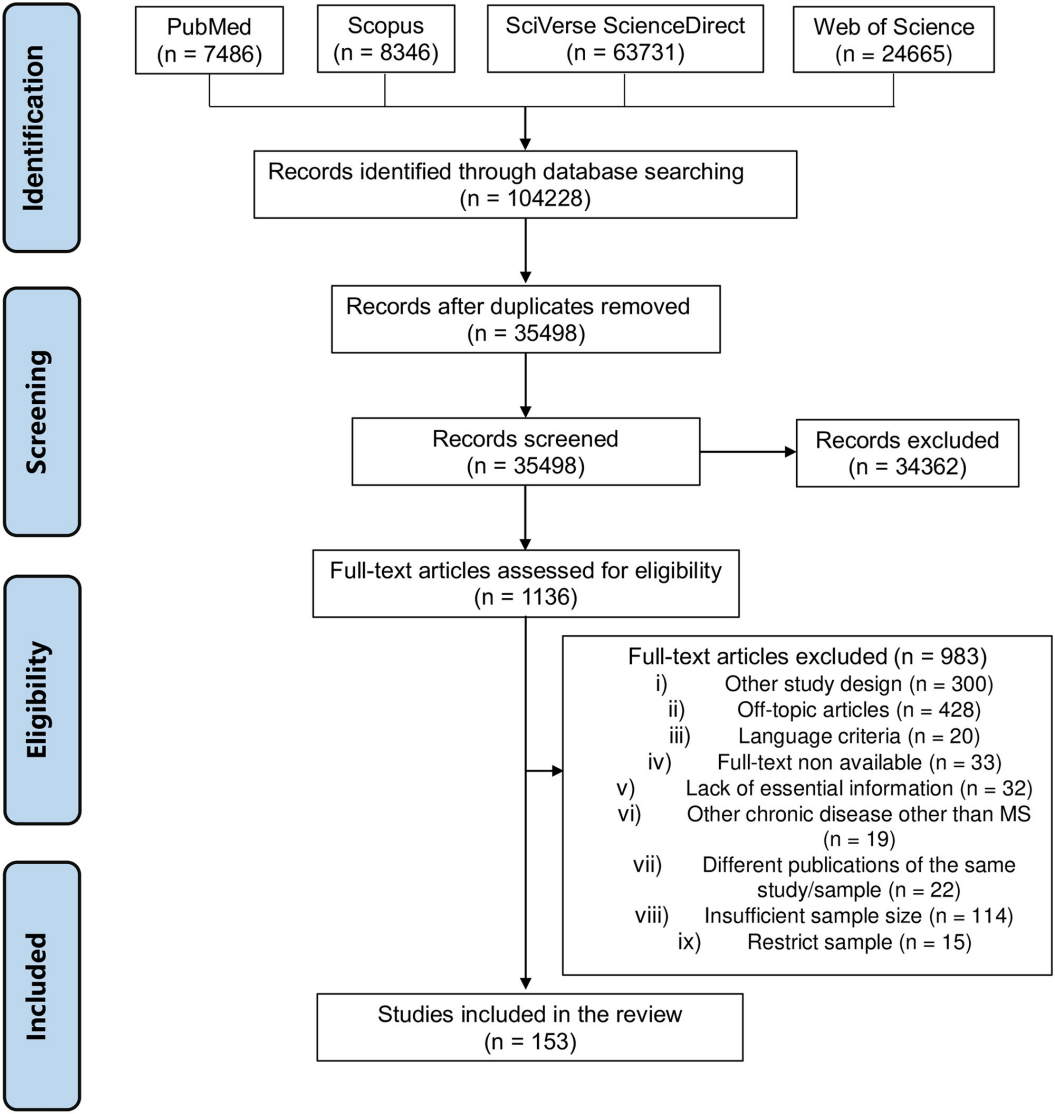
PRISMA flowchart.

Studies written in languages other than the five pre-specified above and studies designed as reviews, letters to the editor, expert opinions, commentaries, case reports, case series, editorials were excluded. In case of articles with missing or dubious data or without an available full text, we tried to contact the corresponding author twice to obtain more information by email. The study was excluded whenever our contact attempt failed. We didn’t accept studies whose sample deliberately included patients with more than a chronic disease or in which MS was not the primary condition. When multiple articles reported data from the same population, the article with the highest number of variables described was selected. We also excluded studies whose study sample were unlikely to be representative of the total population with MS–for example, studies that only focus on specific MS phenotype, only included PwMS with specific deficits or comorbidities, studies that excluded subjects with any disability, or populations primarily selected by the variables of interest. Fig 1 provides the PRISMA flowchart overview of the search and screening strategy performed. Articles were exported and managed in Mendeley 1.19.8 (Elsevier, New York, USA).

### Data analysis and quality assessment

Data extraction was done by two independent reviewers (BKV and AR) and eventual disagreements were resolved by discussion until a consensus was reached. From each eligible study, we extracted the prevalence of unemployment and early retirement. In the cases in which the proportion was not explicit, we calculated it using as the denominator the entire population of the study and as the numerator, the raw explicit number of subjects with any of the outcomes studied. In addition to the outcomes of interest, the following variables were extracted in a Microsoft Excel spreadsheet: name of the first author, year of publication, country, sample size, average age, gender, higher educational attainment (defined as ˃ 12 schooling years), study design, mean Expanded Disability Status Scale (EDSS), mean disease duration, use of disease-modifying drugs (DMDs), MS phenotype (progressive or relapsing-remitting), the prevalence of fatigue, neuropsychiatric symptoms and cognitive impairment. Countries were grouped into continents and were classified by income according to the World Bank country classification 2021 [22]. A data extraction form was used to extract equivalent information in a standardized manner, and to also minimize the intra-examiner variability, all the extracted data were double-checked. Categorical variables are reported as prevalence (%) while numerical variables are reported as means with the respective standard deviation (SD).

Quality assessment of the included studies was carried out using JBI Critical Appraisal Checklist for Studies Reporting Prevalence Data [23]. This checklist was developed to determine the extent to which a prevalence study has addressed the possibility of bias in its design, conduct, and analysis. This questionnaire contains ten closed-ended questions related to the methodological quality of the study. Answers can be "Yes", "No", "Unclear", or "Not/Applicable". The higher the number of "Yes" answers, the higher the quality of the study.

We undertook an initial descriptive analysis of the studies. We used the random-effects model based on the binomial distribution to calculate the pooled estimates of the prevalence of unemployment and early retirement. Specifically, we explored the relationship between these two outcomes with time and geographic variability. When multiple estimates existed for a country and a publication year, these estimates were combined with a random-effects meta-analysis to provide a single estimate for that country or year. We did not use any special statistical treatment for analysing data coming from the same country over time. When at least ten studies presented a specific covariate, we performed a weighted meta-regression with a random-effects model to assess the effect of moderators on the pooled effect size. Differences between the effects-sizes of categorical variables were assessed with the ANOVA Q-Test Random-effects with separate estimates of T2. Effect sizes were reported as proportions. We performed a subgroup analysis by year/time, country, continent, income-based country classification, younger age (< 50 years old), absence of vocational or higher education, disease duration of more than ten years, EDSS greater than 3.0, countries and continents with the highest prevalence of MS (> 200 people with MS per 100.000) [24]. We also did a sensitivity analysis to test the robustness of our findings and we removed possible outliers and studies with a high risk of bias to explore the influence of individual studies on the main results.

Statistical heterogeneity was assessed using the I^2^ statistic and visually inspecting the forest plot. I^2^ more than 75% was regarded as substantial heterogeneity [25]. We investigated the existence of publication bias using Egger’s linear regression test [26], Duval and Tweedie’s Trim and Fill analysis [27], and with the visual inspection of the funnel plots. A p < 0.05 was considered statistically significant. All statistical analyses were performed using ProMeta (version 3.0) and SPSS (version 28.0.1).

## Results

We identified 104228 potentially eligible studies from the systematic search. Removing duplicates and screening the abstracts resulted in 1136 articles whose full-texts were assessed for eligibility. After applying all the inclusion and exclusion criteria, 152 articles were finally considered relevant and included in the qualitative and quantitative analysis (Fig 1). Overall, the total sample size comprised 188436 individuals with MS. The mean age ranged from 32.0 to 60.0 years, the female gender proportion ranged from 33.1 to 100.0%, and the prevalence of individuals with higher educational attainment varied from 24.0% to 88.0%. Concerning the disease characteristics, the mean EDSS and the mean disease duration ranged from 1.3 to 5.5 and 3.2 to 23.6 years, respectively. The proportion of subjects with progressive phenotype of MS varied from 4.6% to 100.0% and the prevalence of fatigue, neuropsychiatric symptoms and cognitive impairment ranged from 56.0% to 96.3%, 25.6% to 89.9%, and 48.1%–97.0%, respectively. Regarding the use of DMDs, there were studies in which no subjects used them and others in which all individuals used them. Data about the prevalence of unemployment and early retirement were available from Data about the prevalence of unemployment and early retirement were available from 151 (99.3%) and 66 (43.5%) studies published from 1981 to 2021, respectively. From the results of the quality assessment, 59 (38.3%) studies were classified as high quality. The minimum data set underlying the results is reported in the S2 Table.

The pooled overall effect size for unemployment was 35.6% (95% CI 32.8–38.4; I^2^ = 99.31). Seven (4.6%) studies resulted in effect sizes greater than 70.0%, with 5 (71.4%) published more than a decade ago [28–34]. Four (2.6%) studies registered effect sizes smaller than 5.0%, of which three were published between 2016 and 2019 [35–38]. Estimates of the prevalence of unemployment ranged from 1.4% to 80.0% (median: 41.1%). The result of the Trim and Fill analysis (p ˂ 0.001), Egger’s linear regression test (p ˂ 0.004), and the visual inspection of the funnel plot (S1 Fig) confirmed the possibility of publication bias.

**Fig 2:**
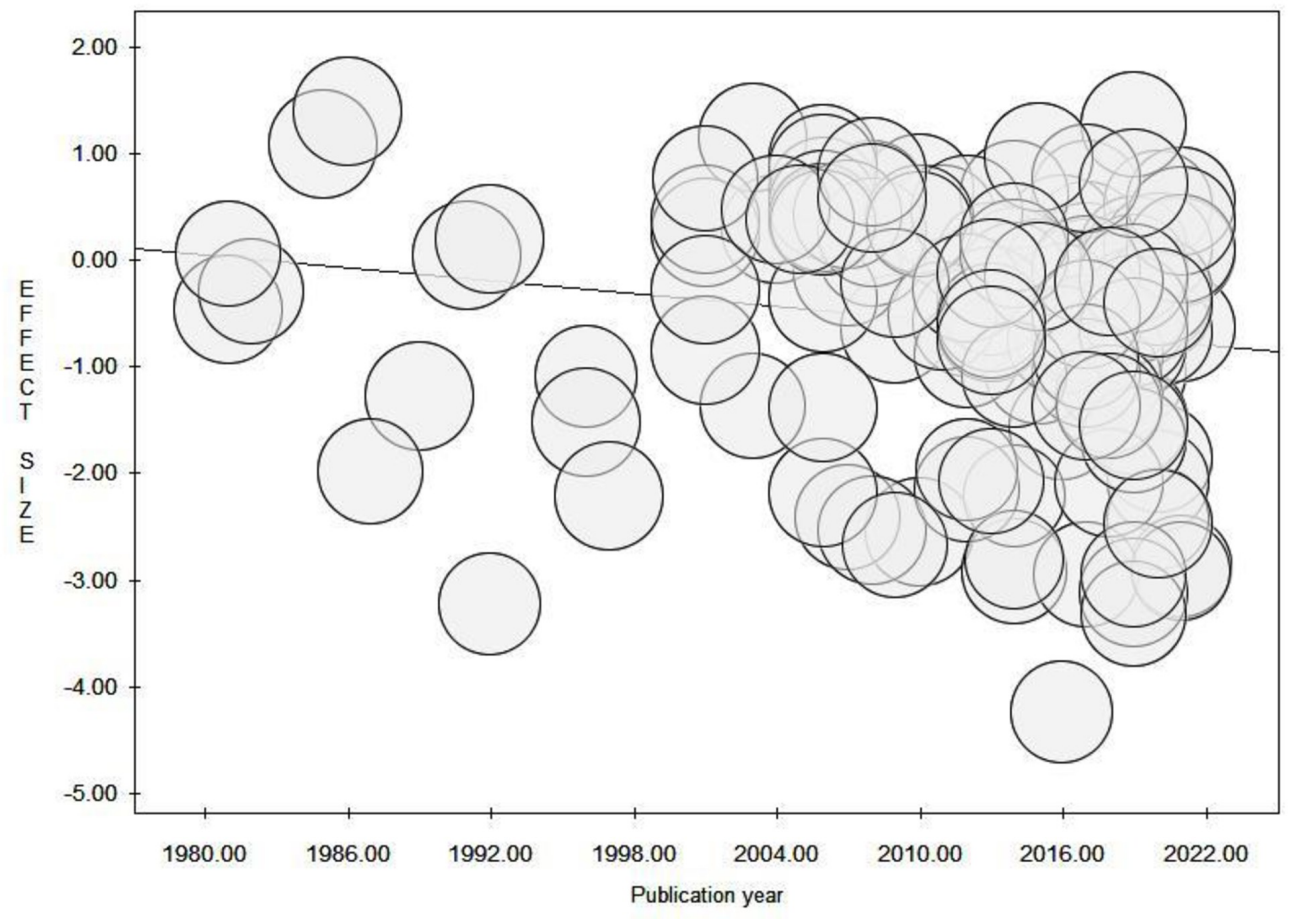
Meta-regression (random-effects model) of the prevalence of unemployment according to time.

**Fig 3:**
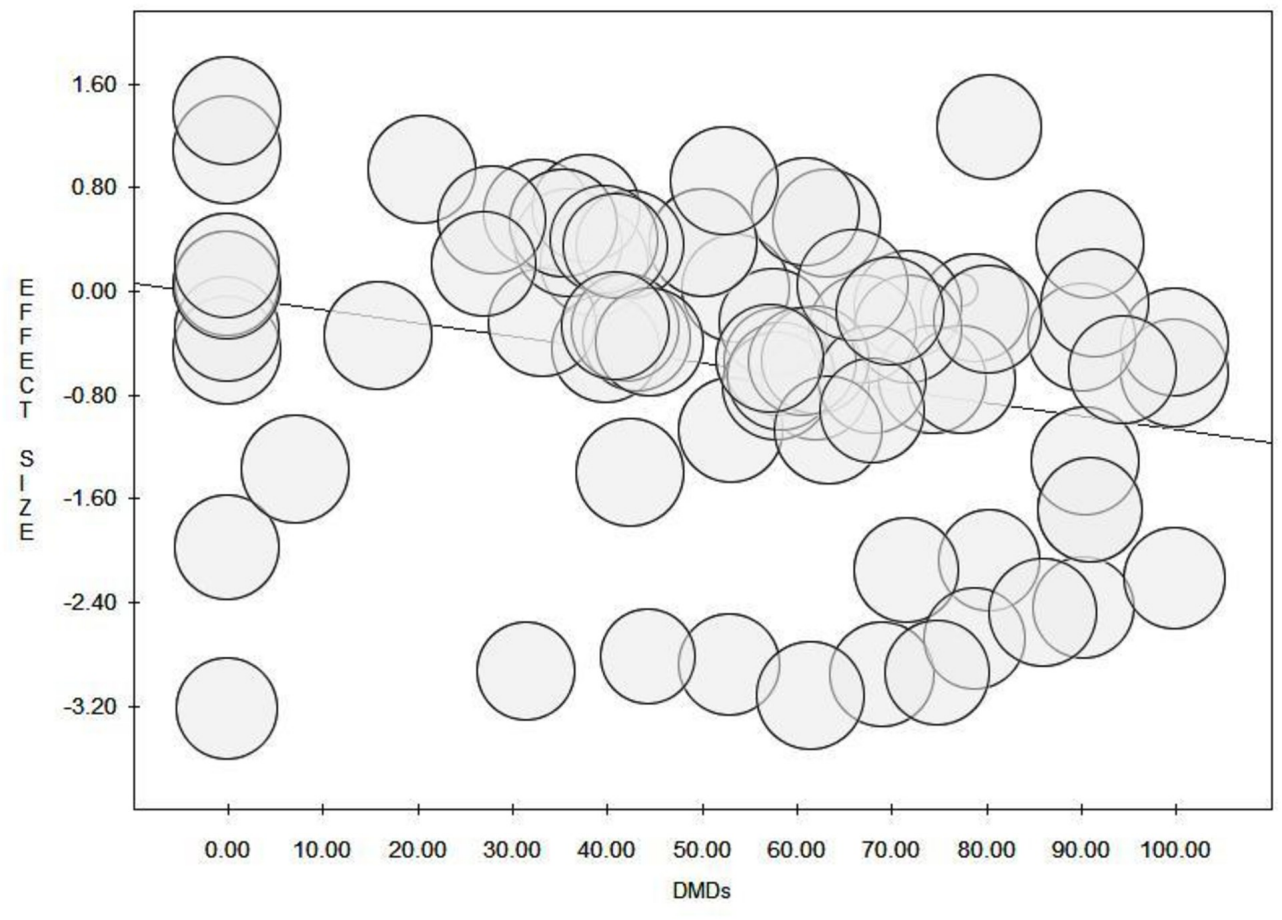
Meta-regression (random-effects model) of the prevalence of unemployment according to the use of DMDs (%).

The prevalence of unemployment varied according to the year of publication (p < 0.001) and there was a statistically significant decrease in the prevalence of unemployment over time (p = 0.042) (Table 1, Fig 2). Globally, the proportion of unemployed subjects with MS remained relatively stable from 1981 to 2010, after which it decreased. The use of DMDs was associated with a reduced prevalence of unemployment (p = 0.021) (Fig 3), especially among those with a longer disease duration (p = 0.035). The decrease in unemployment prevalence over the years was more pronounced among younger individuals and those with a higher EDSS (p = 0.024 and p = 0.010, respectively).

**Table 1:** Effect sizes of prevalence of unemployment by year.

	Effect size	95% CI	Sample size
**1981**	0.44	0.32–0.57	454
**1982**	0.42	0.35–0.49	198
**1985**	0.74	0.69–0.79	312
**1986**	0.79	0.77–0.82	949
**1987**	0.12	0.09–0.15	439
**1989**	0.21	0.18–0.25	508
**1991**	0.50	0.46–0.54	551
**1992**	0.18	0.00–0.86	805
**1996**	0.20	0.14–0.28	532
**1997**	0.09	0.07–0.12	697
**2001**	0.51	0.39–0.62	3884
**2003**	0.46	0.07–0.91	945
**2004**	0.58	0.54–0.62	2149
**2005**	0.59	0.55–0.62	739
**2006**	0.52	0.43–0.61	16816
**2007**	0.33	0.10–0.67	1920
**2008**	0.49	0.33–0.64	17455
**2009**	0.23	0.08–0.48	1101
**2010**	0.42	0.13–0.77	11595
**2011**	0.27	0.11–0.50	2773
**2012**	0.28	0.16–0.45	12813
**2013**	0.34	0.29–0.41	12816
**2014**	0.29	0.21–0.39	14090
**2015**	0.46	0.29–0.64	14922
**2016**	0.23	0.13–0.37	4031
**2017**	0.37	0.29–0.45	27069
**2018**	0.28	0.19–0.38	5823
**2019**	0.27	0.19–0.37	15565
**2020**	0.28	0.17–0.41	7960
**2021**	0.41	0.30–0.52	5136

**Fig 4:**
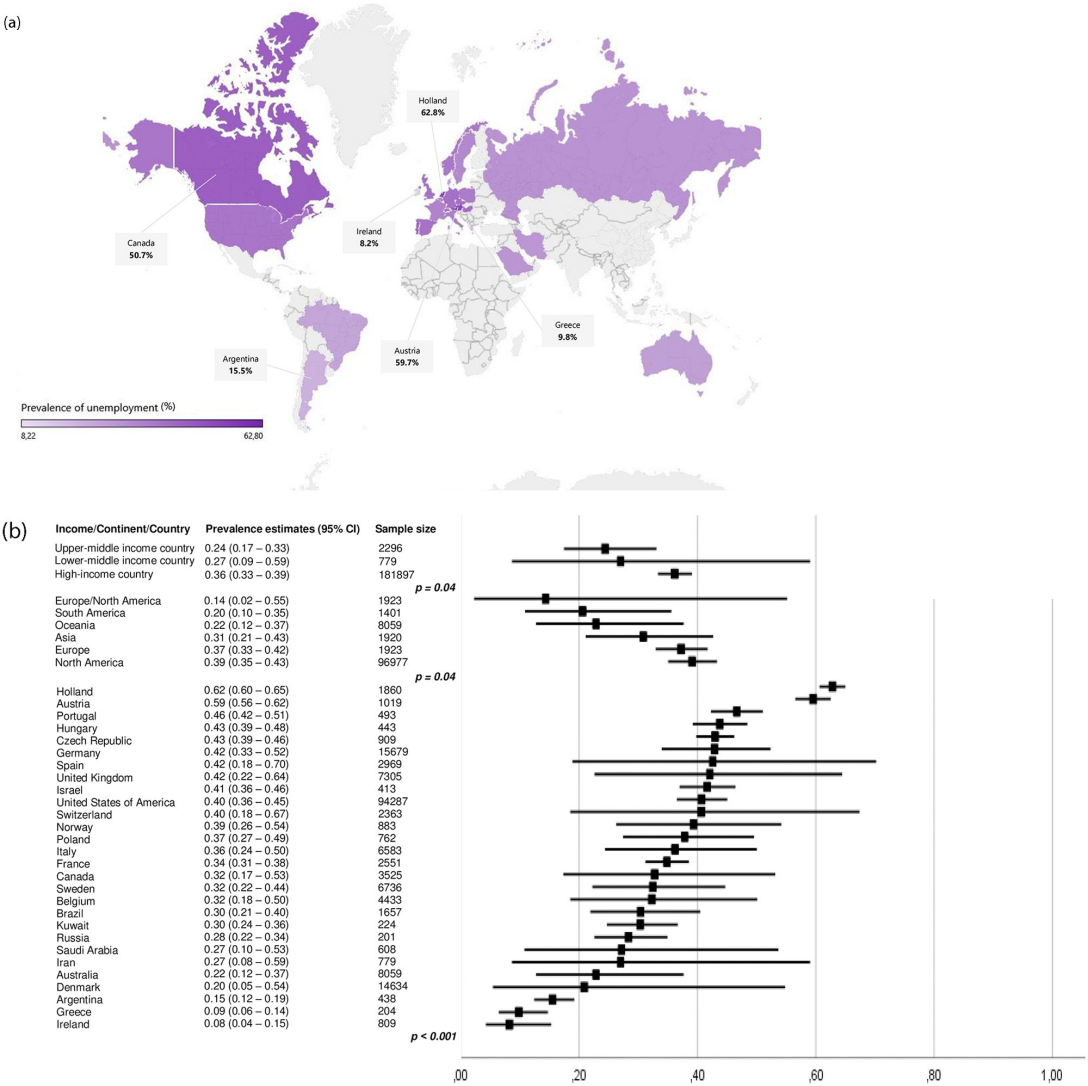
Prevalence of unemployment (%) according to geographical location.

Data concerning the prevalence of unemployment among workers with MS were reported from 29 countries. Austria [39], Czech Republic [40], Greece [41], Hungary [42], Kuwait [43], Portugal [44], Russia [45], and Argentina [46] each contributed with one (0.6%) publication reporting the prevalence of unemployed subjects. Ireland [47, 48], Israel [49, 50], Poland [51, 52], and Saudi Arabia [53, 54] contributed with two (1.3%) studies each while Brazil [55–57], Iran [58–60], Holland [61–63], Norway [11, 64, 65], and Switzerland [66–68] contributed with three (2.0%) studies each. Most of the data on the estimates of the prevalence comes from Canada (four, 2.6%) [69–72], Denmark (five, 3.3%) [73–77], Belgium (five, 3.3%) [35, 78–81], France (five, 3.3%) [82–86], Spain (five, 3.3%) [8, 28, 33, 87, 88], Sweden (seven, 4.6%) [89–95], Germany (eight, 5.3%) [96–103], Australia (eight, 5.9%) [38, 104–110], Italy (nine, 5.9%) [111–119], United Kingdom (nine, 5.9%) [29, 32, 120–126] and the United States of America (50, 32.9%) [14, 15, 30, 31, 34, 36, 37, 127–169]. Five (3.3%) studies were conducted in more than one nation [170–173]. Ireland (8.2%; 95% CI 4.2–15.3) [47, 48], Greece (9.8%; 95% CI 6.4–14.7) [40] and Argentina (15.5%; 95% CI 12.4–19.2) [45, 46] had the best estimates of effect size while Holland (62.8%; 95% CI 60.6–65.0) [61–63], Austria (59.6%; 95% CI 56.5–62.5) [39] and Portugal (46.6%; 95% CI 42.3–51.1) [44] accounted for the highest values. There was a statistically significant difference between the effect sizes of countries (p ˂ 0.001) (Fig 4). From the perspective of continents, 73 (48.0%) studies were performed in Europe, 54 (35.5%) in North America, eight (5.2%) in Asia, eight (5.2%) in Oceania, four (2.6%) in South America and three (2.0%) in Europe and North America. The effect sizes varied in a statistically significant way according to continents (p = 0.04), being North America the continent with the highest pooled prevalence estimate (39.1%; 95% CI 35.1–43.3). Data were provided mostly from high-income countries (142, 93.4%). Upper-middle economy countries and lower-middle economy countries accounted for six (3.9%) and three (2.0%) studies, respectively. The estimates of the prevalence of unemployment significantly varied according to the economic criteria (p = 0.04), being the highest estimate found in high-income economy countries (36.2%; 95% CI 33.3–39.1). Among countries with a high prevalence of MS, a higher educational level was associated with higher proportions of unemployed MS subjects (p = 0.024). In parallel, psychiatric disorders were associated with greater effect sizes of the prevalence of unemployment in Europe (p = 0.046). There was no statistical difference between countries classified according to the prevalence of MS.

The pooled overall effect size for early retirement was 17.2% (95% CI 14.6–20.2; I^2^ = 99.13). In three (4.5%) studies from 2006, 2018, and 2021, the prevalence of early retirement was over 50% [74, 122, 174]. In the most recent studies, the subjects’ sample largely comprised patients who attended rehabilitation clinics or were of advanced age. Five (7.5%) studies accounted for the lowest estimates of early retirement. All were published in 2017 and were conducted in Europe [63, 66, 86, 97, 120]. There was a significant publication bias demonstrated in the funnel plot (S2 Fig), the Trim and Fill analysis (p ˂ 0.001), and the Egger’s linear regression test (p ˂ 0.004).

More than half of the studies included (34, 50.7%) were published in the last seven years (Table 2). Estimates of the prevalence of early retirement ranged from 1.7% to 64.0% (median: 19.2%). Only seven (31.8%) estimates obtained from studies that were published before 2010 were below the overall effect size in comparison to 27 (60.0%) estimates extracted from data published between 2010 and 2021 (p = 0.039). Nevertheless, there was no significant difference between the estimates of the prevalence of early retirement over time on meta-regression analysis (p = 0.082) (S3 Fig), except for the subgroup of younger subjects (p = 0.010) and higher EDSS (p < 0.001). High EDSS and progressive MS phenotype were covariates directly associated with early retirement among younger individuals (p = 0.005 and p = 0.017, respectively). Among those with longer disease duration, the EDSS was a covariate associated with early retirement while among those with higher EDSS, the presence of psychiatric symptoms was the strongest determinant to the observed effect size (p = 0.007).

**Table 2:** Effect sizes of early retirement by year.

	Effect size	95% CI	Sample size
**1981**	0.14	0.01–0.72	454
**1989**	0.15	0.12–0.18	508
**1991**	0.13	0.10–0.16	551
**1992**	0.20	0.11–0.34	814
**2001**	0.33	0.32–0.35	2793
**2006**	0.37	0.34–0.41	16650
**2008**	0.04	0.03–0.41	1942
**2009**	0.15	0.05–0.34	851
**2010**	0.29	0.28–0.31	2538
**2012**	0.30	0.09–0.65	11973
**2013**	0.21	0.06–0.52	9200
**2014**	0.15	0.07–0.29	3550
**2015**	0.07	0.06–0.08	4816
**2016**	0.19	0.09–0.34	2169
**2017**	0.06	0.03–0.11	13786
**2018**	0.17	0.09–0.29	2036
**2019**	0.12	0.07–0.18	5725
**2020**	0.13	0.05–0.28	1574
**2021**	0.63	0.58–0.68	417

**Fig 5:**
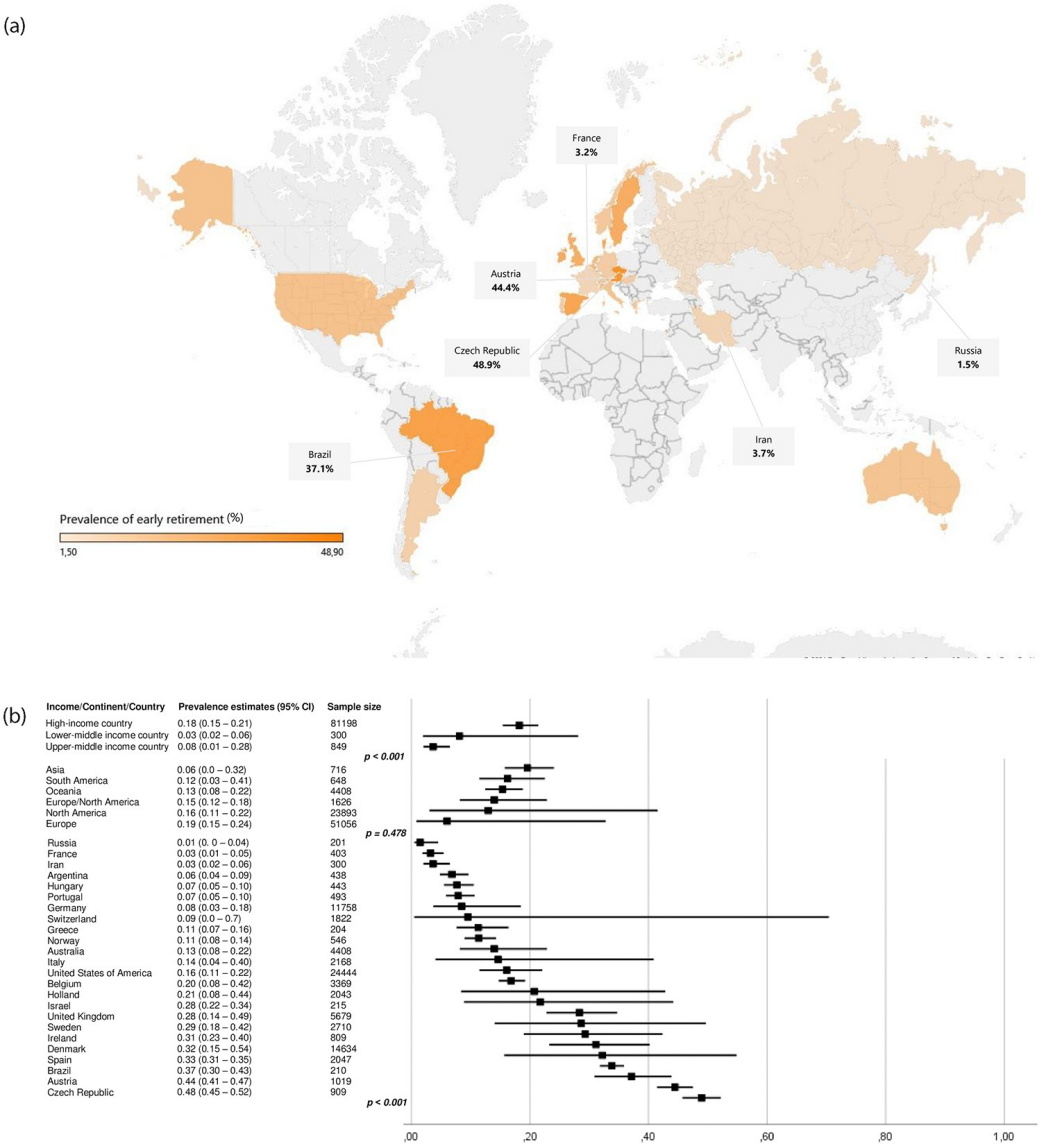
Prevalence of early retirement (%) according to geographical location.

The studies that described the early retirement prevalence were performed in 25 countries: The United States of America (19, 28.3%) [14, 31, 36, 37, 129–132, 136, 140, 143–146, 150, 151, 160, 167, 168] Germany (five, 7.5%) [97, 99, 100, 102, 103] United Kingdom (five, 7.5%) [29, 32, 120, 122, 123], Denmark (four, 6.0%) [73–76], Australia (three, 4.5%) [108, 109, 175], Belgium (three, 4.5%) [35, 79, 80], Holland (three, 4.5%) [61, 63, 176], Italy (three, 4.5%) [111 – 113], Ireland (two, 3.0%) [47, 48], Spain (two, 3.0%) [8, 28], Sweden (two, 3.0%) [90, 91], Switzerland (two, 3.0%) [66, 67], Argentina (one, 1.5%) [46], Austria (one, 1.5%) [39], Brazil (one, 1.5%) [55], the Czech Republic (one, 1.5%) [40], France (one, 1.5%) [85], Greece (one, 1.5%) [41], Hungary (one, 1.5%) [42], Iran (one, 1.5%) [59], Israel (one, 1.5%) [49], Russia (one, 1.5%) [45], Norway (one, 1.5%) [65], and Portugal (one, 1.5%) [44]. One (1.5%) study was multinational [170]. The overall pooled estimate of the prevalence of early retirement was 17.2% (CI 95% 14.6–20.2, I^2^ = 99.13). The three countries with the highest effect sizes were the Czech Republic (48.9%; 95% CI 45.7–52.2) [40], Austria (44.4%; 95% CI 41.4–47.5) [39] and Brazil (37.1%; 95% CI 30.9–43.9) [56] while Russia (1.5%; 95% CI 0.4–4.5) [45], France (3.2%; 95% CI 1.9–5.5) [86] and Iran (3.7%; 95% CI 2.0–6.5) [59] had the lowest proportions (Fig 5). The geographical distribution of the studies is uneven, with Europe (39, 58.2%) and North America (18, 26.9%) accounting for most of the publications. Asia, Oceania, and South America were responsible for three (4.5%) studies each. One study (1.5%) involved both the American and European continents. We couldn’t find any study from Africa. When classifying the countries based on the income criteria, the studies were performed in 62 (92.5%) high-income countries, four (6.0%) upper-middle-income country and only one (1.5%) in a lower-middle-income country. Comparing the effects sizes based on the geographic criteria, there was a substantial difference between countries (p ˂ 0.001) and income-based classified countries (p ˂ 0.001), but no statistically significant difference was found among continents (p ˂ 0.478). We didn’t find any statistical difference between countries classified according to the prevalence of MS but psychiatric disorders were associated with higher estimates of the prevalence of early retirement in countries with high MS prevalence (p = 0.022).

Motor symptoms, gender, cognitive impairment, and fatigue were not associated with any of the outcomes. The sensitivity analysis demonstrated the robustness of our results. Excluding low-quality studies and outliers detected in the funnel plot didn’t show different results for both outcomes.

## Discussion

To the best of our knowledge, this is the first systematic review with a meta-analysis describing the prevalence of unemployment and early retirement in the population of PwMS. The findings confirm the significant negative impact of MS on the occupational environment and the consequently elevated proportions of individuals unemployed and early retired. According to the World Bank Data, between 1991 and 2021, the worldwide unemployment rate ranged from 4.80–6.47%, indicating that workers with MS exceed up to five times the global average estimate [177]. The high prevalence of MS in the world, the manifestation of symptoms at working age, and the presence of several potentially disabling symptoms justify these findings and the particularity of MS at the public health level [66, 178]. These eloquent numbers also give an idea about the effectiveness of the set of actions implemented at the individual and collective level until now, since unemployment and early retirement can be considered sentinels of a spectrum of MS-related outcomes. The diversity of symptoms leaves PwMS vulnerable to a wide variety of barriers at work. In this context, screening PwMS for working difficulties may also prevent or at least postpone unfavourable occupational outcomes. Some questionnaires have already been designed and validated as potential predictors of occupational status change [86]. Moreover, an active investigation of the quality of the integration between worker and work by neurologists and occupational physicians may favour the early recognition of the difficulties and demands of PwMS in the workplace.

We found that there was a decrease in the proportion of workers with MS unemployed over time. This finding has been observed mainly in the last ten years, a period when there was a significant increase in the availability of new DMDs [179]. Indeed, we observed that the use of DMDs was associated with a decrease in the prevalence of unemployment over the years, which is in line with some preliminary evidence [3]. The possibility to control the disease and slow down its progression naturally affects the working capacity of the patient with MS [180]. Consistent with these results, we also found that the association was particularly significant in populations with a high mean EDSS and longer disease duration, important risk factors that are well known to be associated with worse occupational outcomes and that are thought to be extremely influenced by the clinical efficacy of the new MS drugs [13, 118, 151]. The decrease in the prevalence of unemployment was also particularly important among young workers, possibly due to their greater capacity to readjust and engage in new forms of work [174]. Even so, it should be mentioned that the overall observed drop was discrete and can be interpreted as disproportionate to the therapeutic advances in MS and to the time elapsed since the first study reporting the prevalence of unemployment was published. Possible explanations for this lie in the lack of efficient and validated public strategies to promote job retention in workers with MS and the lack of involvement of the occupational physician in this process. Forty percent of patients did not even communicate the diagnosis of MS to their occupational physician [181]. Many of the reasons for work withdrawal are associated with the workplace and could be potentially managed by occupational health multidisciplinary teams [44, 84, 162]. In addition, there is a lack of evidence addressing the reintegration of the worker with MS into the workforce so that nowadays unemployment usually means an irreversible outcome [181], even though almost one-third feel they are still able to work [84]. Regarding specifically early retirement, the results also reinforce the role of MS therapeutic improvement over the years [162]. In addition, aggressive disease characteristics were associated with worse effect estimates of early retirement, which is also consistent with the findings of previous results [182, 183].

We were able include a large number of countries in our analysis. There was a remarkable diversity of estimates of the prevalence of unemployment and early retirement according to the geographical classification. The wide diversity of illness-related unemployment and early retirement across different countries, even from the same continent, is supported by the pre-existing literature for other chronic diseases [184, 185] and supports the argument that occupational outcomes of PwMS are far from depending exclusively on their individual characteristics and are also directly influenced by the public health context. Several national characteristics may explain the differences between the results, so that, based on the global analysis, future studies should be dedicated to better understanding the approach of MS from the occupational viewpoint that justifies the effect sizes of each country. The accurate interpretation of every single result must consider the complexity and particularity of each country’s socio-economic characteristics. A lower unemployment rate at a national level may be associated with larger effects of poor health on not entering employment [162]. Moreover, countries with a high prevalence of unemployment in the general population may also influence the outcomes of PwMS. A general high level of education or a country where there is high competitiveness for highly qualified jobs can explain why some estimates of the prevalence of unemployment are high in Nord America or high-income economy countries. Psychiatric illness may be the most influential clinical manifestation of MS responsible for patients leaving the workforce in countries and continents with a high MS prevalence. It has already been demonstrated that psychiatric illnesses play an important role in the exit of paid employment in Europe [186, 187]. Our study provides preliminary evidence concerning the observation that a higher educational level is associated with greater estimates of the prevalence of unemployment among countries with a high MS prevalence. This finding must be interpreted with caution since factors related to the country’s social, economic and cultural context can have influenced it.

The analyses were made on a large number of subjects from many countries, which strengthens the representativeness of our sample and the quality of the evidence found. We also adopted strict inclusion and exclusion criteria that resulted in a significant proportion of studies with low potential for methodological bias. Besides, our analysis did not include a large number of articles with a high risk of methodological bias. To our knowledge, not only is it the first systematic review with meta-analysis dedicated to analyzing the prevalence of unemployment and early retirement in PwMS, but it is also the first systematic review with meta-analysis to analyze these outcomes in a non-communicable neurological disorder. Our study has also some limitations that need to be acknowledged to allow an accurate interpretation of the results. Although they are relatively simple variables to be measured, by aggregating different types of studies, we could not standardize the way the studies addressed the outcomes, which may be responsible for some kind of methodological bias and the significant heterogeneity. As most of the studies had a cross-sectional design, it is not possible to draw definitive causal relations between the occupational outcomes and MS. We have seen an imbalance in the availability of literature between countries and, therefore, our results might not be representative for some countries or regions. Even though, our study provides a reasonable estimate for countries included in this review, in particular high-income countries. Our study did not include other covariates that could be related to the outcomes. However, our decision was based on which variables were more reported in all studies, preventing the inclusion of insufficient data and the generation of non-significant and unrepresentative data. Finally, we didn’t calculate a score of agreement between the researchers that were responsible for the screening and the selection of articles.

## Conclusions

This systematic review shows that unemployment and early retirement due to MS remain highly prevalent, despite a slight decline in the last decade. This study adds precision and accuracy to the prevalence of unemployment and early retirement in PwMS reported by many previous studies performed in many different countries. Prospective and multicentre cohort studies are encouraged to deepen the knowledge in this field, especially in under-represented countries. The findings should spur more effective public health strategies capable of encompassing the occupational context in which PwMS are inserted to promote their occupational outcomes. Collaboration among clinicians, neurologists, occupational physicians, employers, researchers, and policymakers is urgently required to prevent and mitigate unemployment and early retirement among PwMS.

## Supporting information

S1 File PRISMA checklist. (DOCX)

S1 Table Detailed search strategy in PubMed, Scopus, SciVerse Science Direct and Web of Science. (DOCX)

S2 Table Minimal data set underlying the results and full description of the articles included in the review. NA = Not Applicable. *The JBI Critical Appraisal Checklist for Studies Reporting Prevalence Data was used for the risk of bias assessment, with more stars equalling lower risk.
(DOCX)

S1 Fig Funnel plot of studies included in the analysis (unemployment). (DOCX)

S2 Fig Funnel plot of studies included in the analysis (early retirement). (DOCX)

S3 Fig Meta-regression (random-effects model) of the prevalence of early retirement according to time. (DOCX)
