# Supplementary material for: Spatial and temporal distribution of the prevalence of unemployment and early retirement in people with multiple sclerosis: A systematic review with meta-analysis
Source: PLoS One. 2022 Jul 28;17(7):e0272156. doi: 10.1371/journal.pone.0272156 (PMC9333213; doi:10.1371/journal.pone.0272156)
Supplement: S1 Table — (DOCX) [file pone.0272156.s002.docx]

| PubMed | (Employ* OR unemploy* OR occupation* OR “work” OR vocation* OR “workplace” OR “workforce” OR “labour force” OR “labor force” OR Career* OR Job* OR retire* OR “disability pension” OR “worker” OR “fitness for work”) AND (“Multiple sclerosis” OR “Demyelinating Autoimmune Diseases” OR “Demyelinating Autoimmune Disorders” OR “Clinically Isolated Syndrome” OR “Demyelinating”) |
| --- | --- |
| Scopus | TITLE-ABS KEY [(employ* OR unemploy* OR occupation* OR "work" OR vocation* OR  “workplace” OR "workforce" OR "labour force" OR "labor force" OR career* OR job* OR "job retention" OR retire* OR "disability pension" OR "worker" OR "fitness for work") AND ("Multiple sclerosis" OR "Demyelinating Autoimmune Diseases" OR "Demyelinating Autoimmune Disorders" OR "Clinically Isolated Syndrome" OR "Demyelinating")] |
| SciVerse Science Direct | ("Employ" OR "occupation" OR "work" OR "vocation" OR “labour” OR "Job" OR  "retire" OR “disability pension”) AND “Multiple sclerosis” |
| Web of Science | (Employ* OR unemploy* OR occupation* OR “work” OR vocation* OR “workplace” OR “workforce” OR “labour force” OR “labor force” OR Career* OR Job* OR retire* OR  “disability pension” OR “worker” OR “fitness for work”) AND (“Multiple sclerosis” OR “Demyelinating Autoimmune Diseases” OR “Demyelinating Autoimmune Disorders”  OR “Clinically Isolated Syndrome” OR “Demyelinating”) |
