# Supplementary material for: Spatial and temporal distribution of the prevalence of unemployment and early retirement in people with multiple sclerosis: A systematic review with meta-analysis
Source: PLoS One. 2022 Jul 28;17(7):e0272156. doi: 10.1371/journal.pone.0272156 (PMC9333213; doi:10.1371/journal.pone.0272156)
Supplement: S2 Table — NA = Not Applicable. *The JBI Critical Appraisal Checklist for Studies Reporting Prevalence Data was used for the risk of bias assessment, with more stars equalling lower risk. (DOCX) [file pone.0272156.s003.docx]

| Authors | Country | Year | Study design | N | Mean age (SD) | Female sex (%) | Educational level ˃ 12 years (%) | EDSS (Mean) | Disease duration, years (Mean) | Progressive MS (%) | Treatment (%) | N_E (%) | N_R (%) | JBI quality assesment |
| --- | --- | --- | --- | --- | --- | --- | --- | --- | --- | --- | --- | --- | --- | --- |
| Alhussain et al.^1^ | Saudi Arabia | 2020 | Cross-sectional | 238 | 32 (7.9) | 60.9 | 61.8 | NA | 7.1 (4.7) | NA | NA | 39.9 | NA | ******** |
| AlZahrani^2^ | Saudi Arabia | 2019 | Cross-sectional | 370 | NA | 67.3 | 53.2 | NA | NA | NA | NA | 17.3 | NA | ******* |
| Aronson et al.^3^ | Canada | 1997 | Cross-sectional | 697 | 48 (NA) | 70.0 | NA | NA | NA | NA | NA | 68.0 | NA | ********* |
| Arroyo et al.^4^ | Spain | 2013 | Cross-sectional | 409 | 46.4 (11.0) | 62.4 | NA | NA | 12,5 | 57.9 | NA | 11.0 | NA | ****** |
| Bamer et al.^5^ | United States of America | 2008 | Cross-sectional | 1268 | 50.5 (11.3) | 76.8 | 32.8 | NA | 13,5 | 50.6 | NA | 63.8 | NA | ******* |
| Baroin et al.^6^ | France | 2013 | Cross-sectional | 305 | 41 (9.8) | 77.0 | 58.3 | 2.2 (1.7) | 7,4 | 92.6 | NA | 31.8 | NA | ****** |
| Bass et al.^7^ | Multinational | 2020 | Cross-sectional | 1075 | NA | NA | NA | NA | NA | NA | 86.0 | 7.7 | 16.9 | ******** |
| Battaglia et al.^8^ | Italy | 2017 | Cross-sectional | 950 | 45.0 (12.0) | 70.0 | 30.0 | 3.7 (2.6) | NA | 33.0 | 7.3 | 20.0 | 4.0 | ********* |
| Baumstarck et al^9^. | France | 2013 | Longitudinal study | 526 | 40.0 (9.9) | 74.3 | 67.9 | 2.5 (1.7) | NA | NA | 94.5 | 35.2 | NA | ******** |
| Becker et al.^10^ | Germany | 2018 | Cross-sectional case-control | 2791 | 41.4 (11.0) | 72.5 | NA | NA | NA | NA | NA | 44.8 | NA | ******* |
| Beier et al.^11^ | United States of America | 2019 | Cross-sectional case-control | 407 | 53.9 (9.8) | 83.0 | 47.4 | NA | 16.6 (9.1) | 40.5 | NA | 66.8 | NA | ******** |
| Berg et al.^12^ | Sweden | 2006 | Cross-sectional case-control | 1339 | 53.4 (12.0) | 73.0 | 61.1 | 5.1 (2.2) | 14.0 (NA) | 67.7 | 42.6 | 19.8 | 35.7 | ********* |
| Bishop et al. ^13^ | United States of America | 2009 | Cross-sectional | 250 | 43.0 (9.0) | 81.0 | 78.0 | NA | 4.2 (3.8) | NA | 80.3 | 44.5 | NA | ******* |
| Bishop et al. ^14^ | United States of America | 2009 | Cross-sectional | 409 | 44.5 (10.9) | 84.0 | NA | NA | 5.3 (6.9) | 58.2 | 78.9 | 6.3 | 9.5 | ******* |
| Bishop et al. ^15^ | United States of America | 2013 | Cross-sectional | 4201 | 52.0 (8.7) | 79.3 | 59.7 | NA | 14.8 (8.5) | 85.2 | NA | 46.6 | 7.6 | ******** |
| Bishop et al. ^16^ | United States of America | 2015 | Cross-sectional | 615 | 51.9 (NA) | 79.1 | 59.9 | NA | NA | NA | 69.6 | 45.9 | 7.7 | ****** |
| Bishop et al. ^17^ | United States of America | 2019 | Cross-sectional | 748 | 52.4 (12.1) | 72.7 | NA | NA | NA | 37.0 | 75.0 | 5.0 | 10.0 | ****** |
| Bo et al.^18^ | Italy | 2018 | Cross-sectional | 297 | 49.5 (10.7) | 71.4 | 18.5 | 3.6 (2.5) | NA | 27.0 | NA | 20.2 | 19.2 | ****** |
| Bøe Lunde et al.^19^ | Norway | 2014 | Cross-sectional | 213 | NA | 69.0 | 29.1 | 4.0 (3.0) | 18.9 (11.5) | 81.1 | 27.1 | 54.9 | NA | ********* |
| Boyko et al.^20^ | Russia | 2017 | Cross-sectional | 201 | 38.5 (10.5) | 65.0 | 64.0 | 2.9 (2.1) | NA | 42.8 | 68.1 | 28.3 | 1.5 | ********* |
| Broersma et al.^21^ | Holland | 2018 | Cross-sectional | 185 | 60.0 (10.8) | 68.0 | 24.0 | NA | 23.6 (8.4) | 51.0 | NA | NA | 52.0 | ***** |
| Brundin et al.^22^ | Sweden | 2017 | Cross-sectional | 1371 | 56.2 (12.0) | 78.3 | 55.0 | 4.7 (2.6) | NA | NA | 57.2 | 37.3 | 23.6 | ********* |
| Buchanan et al. ^23^ | United States of America | 2008 | Cross-sectional | 4943 | 58.7 (9.9) | 63.2 | 73.9 | NA | 19.6 (10.7) | 100.0 | NA | 69.4 | NA | ***** |
| Buhse et al.^24^ | United States of America | 2014 | Cross-sectional | 211 | NA | NA | NA | NA | NA | NA | NA | 25.0 | NA | ***** |
| Calabrese et al. ^25^ | Switzerland | 2017 | Longitudinal cohort study | 721 | 48.4 (11.9) | 74.3 | 23.4 | 3.1 (2.5) | NA | 39.4 | 63.5 | 28.2 | 2.1 | ********* |
| Carnero Contentti^26^ | Argentina | 2019 | Cross-sectional | 219 | 39.0 (16.0) | 68.0 | 48.6 | 2.5 (2.0 | NA | 4.6 | 91.0 | 15.5 | 6.8 | ******** |
| Carney et al.^27^ | Ireland | 2018 | Cross-sectional | 595 | NA | 71.4 | NA | NA | 14.7 (NA) | 37.1 | NA | 11.0 | 27.3 | ******** |
| Catanzaro et al.^28^ | United States of America | 1992 | Cross-sectional | 604 | NA | NA | 24.0 | NA | 10.0 | NA | 0.0 | 1.9 | 13.4 | ******** |
| Chamot et al.^29^ | United States of America | 2014 | Cross-sectional | 7851 | 46.0 (11.1) | 80.0 | NA | NA | 15.0 (11.3) | NA | 66.0 | 51.0 | NA | ******** |
| Chen et al. ^30^ | Australia | 2018 | Cross-sectional | 874 | 49.7 (9.7) | 82.0 | 47.2 | NA | 11.5 (6.4) | 18.5 | 90.6 | 21.6 | NA | ********* |
| Chen et al. ^31^ | Australia | 2019 | Cross-sectional | 740 | 51.1 (8.8) | 80.6 | 38.7 | NA | 12.4 (7.0) | 30.2 | NA | 3.5 | NA | ********* |
| Chen et al. ^32^ | Australia | 2019 | Longitudinal cohort study | 1240 | NA | 77.8 | 40.7 | NA | NA | 32.9 | 68.1 | 33.3 | NA | ********* |
| Chiu et al.^33^ | United States of America | 2012 | Cross-sectional | 215 | 47.4 (10.2) | 86.0 | 34.0 | NA | NA | NA | NA | 12.1 | 39.1 | ******* |
| Chiu et al.^34^ | United States of America | 2015 | Longitudinal cohort study and case-control study | 8715 | NA | 68.0 | NA | NA | NA | NA | NA | 72.0 | NA | ********* |
| Chiu et al.^35^ | United States of America | 2019 | Cross-sectional | 3003 | 58.4 (11.7) | 82.9 | 66.1 | NA | NA | 45.2 | 61.6 | 4.2 | 27.6 | ******** |
| Chwastiak et al.^36^ | United States of America | 2005 | Cross-sectional | 739 | NA | 77.9 | 67.4 | NA | NA | NA | NA | 59.2 | NA | ******* |
| Clingerman et al.^37^ | United States of America | 2004 | Longitudinal cohort study | 839 | 47.0 (10.1) | 100.0 | 66.2 | NA | 10.3 (7.4) | NA | NA | 61.2 | NA | ********* |
| Cofield et al.^38^ | United States of America | 2017 | Cross-sectional | 7009 | 57.6 (10.3) | 79.3 | NA | NA | 19.1 (9.8) | 43.6 | NA | 67.9 | NA | ********* |
| Coleman et al.^39^ | United States of America | 2013 | Cross-sectional | 3728 | NA | 80.1 | NA | NA | NA | NA | 60.8 | 36.6 | 43.7 | ********* |
| da Silva et al.^40^ | Brazil | 2016 | Cross-sectional | 210 | 40.7 (11.5) | 70.0 | 34.7 | NA | 7.9 (6.2) | 92.1 | NA | 17.1 | 37.0 | ******** |
| D'hooghe et al.^41^ | Belgium | 2016 | Cross-sectional | 1353 | NA | NA | 42.5 | NA | 19.3 (NA) | 80.7 | NA | 1.4 | 12.1 | ******** |
| D'hooghe et al.^42^ | Belgium | 2019 | Cross-sectional | 751 | 53.4 (NA) | 66.2 | NA | 4.8 (NA) | 20.3 (NA) | NA | NA | 61.1 | NA | ******** |
| Dubois et al.^43^ | Belgium | 2017 | Cross-sectional | 1217 | 54.0 (12.6) | 70.0 | 64.0 | 4.6 (2.5) | NA | 57.0 | 91.5 | 47.5 | 15.1 | ********* |
| Dusankova et al.^44^ | Czech Republic | 2012 | Cross-sectional | 909 | 41.0 (12.0) | NA | NA | NA | 8.0 (8.0) | 34.0 | 41.0 | 43.0 | 49.0 | ********* |
| Esposito et al.^45^ | Italy | 2021 | Cross-sectional | 435 | 42.4 (11.4) | 68.4 | NA | NA | NA | 18.7 | 91.1 | 40.9 | NA | ******* |
| Estrutti et al.^46^ | Brazil | 2019 | Cross-sectional | 753 | 36.2 (NA) | 84.0 | 67.0 | NA | NA | NA | NA | 38.2 | NA | ****** |
| Fantoni-Quinton et al.^47^ | France | 2016 | Cross-sectional | 941 | 46.1 (9.8) | 79.8 | NA | NA | 10.3 (7.3) | NA | NA | 31.9 | NA | ****** |
| Farnoush et al.^48^ | Iran | 2010 | Cross-sectional | 174 | NA | 71.3 | 46.6 | NA | NA | NA | NA | 58.0 | NA | ***** |
| Fernandez et al.^49^ | Multinational | 2011 | Cross-sectional | 1992 | 42.3 (12.5) | 70.5 | 35.2 | 3.2 (2.6) | 11.8 (8.8) | 88.2 | 62.1 | 36.8 | NA | ******* |
| Fernandez-Jimenez et al.^50^ | Multinational | 2021 | Cross-sectional | 297 | 40.5 (8.1) | NA | NA | 3.1 (1.6) | 8.5 (5.9) | 67.0 | 31.6 | 5.0 | NA | ******* |
| Fidao et al.^51^ | Australia | 2021 | Cross-sectional | 2104 | 45.7 (10.5) | 82.5 | NA | NA | NA | 23.8 | NA | 44.8 | NA | ********* |
| Flachenecker et al.^52^ | Germany | 2017 | Cross-sectional | 5475 | 51.8 (11.0) | 74.3 | 55.0 | 4.0 (2.5) | NA | 54.0 | 58.5 | 34.0 | 1.8 | ********* |
| Flensner et al.^53^ | Sweden | 2013 | Cross-sectional | 323 | 47.5 (10.8) | 76.3 | 27.2 | NA | NA | 21.7 | 44.6 | 40.2 | NA | ********* |
| Fogarty et al.^54^ | Ireland | 2014 | Cross-sectional | 214 | 47.6 (12.7) | NA | NA | 3.6 (2.6) | 14.8 (10.8) | 85.2 | 44.4 | 6.1 | 36.0 | ******* |
| Forbes et al.^55^ | United Kingdom | 2006 | Cross-sectional | 929 | 48.0 (11.4) | 69.0 | NA | NA | 16.0 (10.4) | 55.0 | NA | 10.0 | 52.0 | ******** |
| Ford et al. ^56^ | United Kingdom | 2001 | Cross-sectional | 203 | 46.0 (NA) | 78.0 | NA | NA | 14.4 (0.7) | NA | NA | 68.0 | NA | ********* |
| Garcia-Dominguez et al.^57^ | Spain | 2019 | Cross-sectional | 199 | 43.9 (10.5) | 60.8 | 39.7 | 2.2 (1.9) | 9.6 (7.2) | 13.6 | 80.4 | 52.8 | NA | ******* |
| Genevie et al.^58^ | United States of America | 1987 | Cross-sectional | 439 | NA | 75.0 | 55.0 | NA | 11.0 (NA) | NA | 0.0 | 12.0 | NA | ****** |
| Gil-Gonzàlez et al.^59^ | Spain | 2021 | Longitudinal cohort study | 314 | 45.3 (10.7) | 67.8 | 53.5 | 3.2 (1.9) | 12.1 (7.5) | 13.4 | 28.0 | 63.1 | NA | ******* |
| Glanz et al.^60^ | United States of America | 2012 | Longitudinal cohort study | 377 | 45.4 (10.6) | 76.1 | 7.8 | 1.3 (1.9) | 12.4 (7.8) | 87.6 | 71.7 | 10.3 | NA | ********* |
| Gottberg et al.^61^ | Sweden | 2006 | Cross-sectional | 166 | 51.0 (12.0) | 71.0 | 40.0 | NA | NA | 58.0 | 41.0 | 59.0 | NA | ******* |
| Gross et al.^62^ | United States of America | 2017 | Cross-sectional | 810 | 50.2 (12.0) | 47.8 | 26.8 | NA | NA | 33.0 | 69.1 | 4.9 | 15.5 | ******** |
| Gulick et al.^63^ | United States of America | 1989 | Cross-sectional | 508 | 48.1 (8.9) | 33.1 | NA | NA | 13.6 (8.2) | NA | 0.0 | 21.6 | 15.5 | ***** |
| Gulick et al.^64^ | United States of America | 1991 | Longitudinal cohort study | 551 | 49.7 (11.5) | 74.0 | NA | NA | 14.6 (9.1) | NA | 0.0 | 50.9 | 13.6 | ****** |
| Gulick et al.^65^ | United States of America | 1992 | Cross-sectional | 201 | 47.4 (11.6) | 75.0 | NA | NA | 11.0 (8.4) | NA | 0.0 | 55.2 | 15.7 | **** |
| Gulick et al.^66^ | United States of America | 1996 | Cross-sectional | 408 | 45.8 (4.5) | 100.0 | NA | NA | 11.7 (7.0) | NA | NA | 11.5 | NA | ******* |
| Hadjigeorgiou et al. ^67^ | Greece | 2014 | Cross-sectional | 204 | 43.6 (NA) | 70.0 | 54.9 | NA | 7.5 (6.4) | NA | 100.0 | 9.8 | 11.3 | ******* |
| Hategeka et al.^68^ | Canada | 2019 | Cross-sectional | 530 | NA | 74.9 | 65.7 | NA | 14.4 (1.2) | NA | NA | 47.8 | NA | ******* |
| Heiznlef et al.^69^ | France | 2020 | Cross-sectional | 376 | 48.3 (NA) | 76.9 | 46.3 | NA | NA | 52.0 | 71.8 | 42.6 | NA | ****** |
| Henriksson et al.^70^ | Sweden | 2001 | Cross-sectional | 413 | 49.0 (12.3) | 71.0 | NA | 4.9 (NA) | 17.1 (NA) | 66.0 | 42.0 | 43.0 | NA | ******** |
| Honan et al.^71^ | Australia | 2014 | Cross-sectional | 189 | NA | 74.1 | NA | NA | NA | NA | NA | 46.0 | NA | ******** |
| Iezzoni et al.^72^ | United States of America | 2007 | Cross-sectional | 983 | NA | 78.9 | 71.5 | NA | NA | 27.3 | NA | 60.2 | NA | ********* |
| Iezzoni et al.^73^ | United States of America | 2010 | Cross-sectional | 703 | NA | 77.5 | 40.0 | NA | NA | NA | NA | 59.1 | NA | ********* |
| Imani et al.^74^ | Iran | 2020 | Cross-sectional | 300 | 37.1 (9.7) | NA | NA | NA | 9.9 | NA | 90.3 | 8.0 | 3.7 | ******** |
| Iwanaga et al.^75^ | United States of America | 2018 | Cross-sectional case-control | 154 | 41.0 (8.4) | 87.0 | 88.0 | NA | NA | 18.1 | NA | 24.6 | 5.8 | ******* |
| Jennum et al.^76^ | Denmark | 2012 | Cross-sectional | 10849 | NA | 66.0 | NA | NA | NA | NA | NA | 61.0 | 12.0 | ********* |
| Johansson et al.^77^ | Denmark | 2020 | Longitudinal cohort study | 264 | NA | 68.0 | NA | NA | NA | 44.0 | 67.0 | 42.0 | NA | ******* |
| Johansson et al.^78^ | Denmark | 2021 | Cross-sectional | 417 | 54.8 (2.2) | NA | 23.0 | NA | 8.0 (2.0) | 59.0 | 53.0 | 5.5 | 64.0 | ******* |
| Johnson et al.^79^ | United States of America | 2013 | Cross-sectional | 1271 | 48.6 (9.9) | 80.8 | 48.4 | NA | 12.0 (9.0) | 88.0 | NA | 34.6 | 25.4 | ********* |
| Jones et al.^80^ | United States of America | 2016 | Cross-sectional | 715 | 42.1 (10.7) | 69.3 | NA | NA | 6.9 (5.3) | NA | 90.2 | 17.1 | NA | ****** |
| Julian et al.^81^ | United States of America | 2008 | Cross-sectional AND longitudinal cohort study | 8867 | 47.6 (8.6) | 75.0 | 34.6 | NA | 20.3 (10.2) | NA | NA | 56.2 | NA | ********* |
| Kalantari et al.^82^ | Iran | 2018 | Cross-sectional | 305 | 32.0 (9.1) | 74.8 | NA | NA | 7.4 (5.7) | NA | NA | 29.2 | NA | ******* |
| Kallmann et al.^83^ | Germany | 2019 | Longitudinal cohort study | 1128 | 44.9 (10.2) | 67.5 | NA | 2.3 (1.5) | 8.9 (7.6) | NA | 100.0 | 40.0 | NA | ******* |
| Khader et al.^84^ | Kuwait | 2019 | Cross-sectional | 224 | NA | NA | 67.9 | NA | NA | NA | NA | 30.4 | NA | ****** |
| Kirk-Brown et al.^85^ | Australia | 2014 | Longitudinal cohort study | 673 | 46.7 (9.2) | 83.0 | NA | NA | NA | NA | NA | 5.0 | 5.0 | ********* |
| Kobelt et al.^86^ | Germany | 2001 | Cross-sectional | 2793 | 45.1 (11.1) | 72.2 | NA | 3.8 (2.3) | 10.1 (NA) | 60.3 | 50.3 | 59.0 | 33.9 | ********* |
| Kobelt et al.^87^ | Austria | 2006 | Cross-sectional | 1019 | 50.0 (12.2) | 70.4 | 31.1 | 4.4 (2.4) | 15.3 (NA) | NA | 39.8 | 59.6 | 44.5 | ********* |
| Kobelt et al.^88^ | Belgium | 2006 | Cross-sectional | 799 | 46.7 (NA) | 69.1 | 50.8 | 3.9 (2.2) | 9.7 (NA) | NA | 35.5 | 62.6 | 42.2 | ********* |
| Kobelt et al.^89^ | United Kingdom | 2006 | Cross-sectional | 2048 | 51.4 (10.7) | 74.5 | NA | NA | 12.6 (NA) | NA | 20.6 | 71.8 | 44.3 | ********* |
| Kobelt et al.^90^ | Holland | 2006 | Cross-sectional | 1549 | 46.7 (NA) | 69.5 | 50.8 | 3.9 (2.2) | 9.7 (NA) | NA | 35.5 | 62.6 | 42.2 | ********* |
| Kobelt et al.^91^ | Italy | 2006 | Cross-sectional | 921 | 46.1 (11.7) | 65.8 | 60.5 | 4.6 (2.3) | 12.8 (NA) | NA | 42.6 | 58.9 | 33.3 | ********* |
| Kobelt et al.^92^ | Spain | 2006 | Cross-sectional | 1848 | 44.7 (NA) | 64.2 | 30.5 | 4.5 (2.3) | 11.7 (NA) | NA | 52.4 | 70.0 | 34.1 | ********* |
| Kobelt et al.^93^ | Switzerland | 2006 | Cross-sectional | 1101 | 52.7 (NA) | 63.8 | 68.6 | 4.6 (2.4) | 16.3 (NA) | NA | 37.9 | 65.3 | 33.9 | ********* |
| Kobelt et al.^94^ | Germany | 2006 | Cross-sectional | 2973 | 45,1 (NA) | 72.2 | 70.0 | 3.8 (2.3) | 10.1 (NA) | NA | 50.3 | 59.1 | 33.1 | ********* |
| Kobelt et al.^95^ | United States of America | 2006 | Cross-sectional | 1909 | 49.2 (9.5) | 76.4 | NA | NA | 13.1 (8.1) | NA | NA | 59.1 | 31.4 | ********* |
| Selmaj et al.^96^ | Poland | 2017 | Cross-sectional | 387 | 39.7 (12.3) | 74.0 | 59.0 | 3.5 (2.3) | NA | 36.0 | 58.0 | 32.3 | NA | ********* |
| Kobelt et al.^97^ | Brazil | 2019 | Cross-sectional | 694 | 40.8 (11.3) | NA | 62.1 | 3.2 (2.5) | NA | 25.2 | 58.1 | 38.7 | NA | ********* |
| Koch et al.^98^ | United States of America | 2001 | Cross-sectional | 227 | 47.0 (11.7) | 71.0 | 64.7 | NA | NA | 28.0 | NA | 56.0 | NA | ****** |
| Kohn et al.^99^ | United States of America | 2014 | Cross-sectional | 3225 | NA | 80.1 | NA | NA | NA | NA | 61.0 | 64.7 | NA | ********* |
| Kornblith et al.^100^ | United States of America | 1986 | Cross-sectional | 949 | 48.3 (NA) | 67.2 | NA | NA | 12.5 (NA) | NA | 0.0 | 80.0 | NA | ******** |
| Kos et al.^101^ | Belgium | 2020 | Cross-sectional | 313 | 49.6 (10.3) | 67.4 | NA | NA | NA | NA | NA | 52.7 | NA | ****** |
| Krause et al.^102^ | United States of America | 2019 | Cross-sectional | 1059 | 48.0 (10.3) | 77.8 | NA | NA | NA | NA | NA | 51.9 | NA | ****** |
| Krause et al.^103^ | United States of America | 2021 | Cross-sectional | 1234 | NA | 77.6 | 50.4 | NA | NA | NA | NA | 51.6 | NA | ******** |
| Krokavkova et al.^104^ | Multinational | 2008 | Cross-sectional | 203 | 38.3 (10.6) | 64.3 | 51.7 | 3.0 (1.5) | 5.3 (4.1) | 27.7 | NA | 65.5 | NA | ****** |
| LaRocca et al.^105^ | United States of America | 1985 | Cross-sectional | 312 | 43.0 (NA) | NA | NA | 4.6 (NA) | NA | NA | 0.0 | 77.0 | NA | ****** |
| Lebrun-Fernay et al.^106^ | France | 2017 | Cross-sectional | 403 | 47.2 (13.1) | 74.0 | 53.0 | 3.6 (2.4) | NA | 39.0 | 77.5 | 33.5 | 3.2 | ********* |
| Lehmann et al.^107^ | Switzerland | 2020 | Cross-sectional | 541 | 48.0 (2.5) | 76.7 | 55.0 | NA | 10.0 (1.8) | 24.6 | 74.5 | 33.4 | NA | ******* |
| Li et al.^108^ | United States of America | 2015 | Cross-sectional | 4201 | 52.0 (8.7) | 79.3 | 59.7 | NA | NA | NA | NA | 46.6 | 7.6 | ********* |
| Marck et al.^109^ | Australia | 2019 | Longitudinal cohort study | 1276 | 45.1 (9.1) | 83.7 | 79.6 | NA | NA | NA | NA | 18.0 | 19.7 | ******* |
| Maurino et al.^110^ | Spain | 2020 | Cross-sectional | 199 | 43.9 (10.5) | 60.8 | 39.7 | 2.5 (2.0) | 9.6 (7.1) | 13.6 | 80.4 | 11.1 | 31.2 | ******* |
| McCrone et al.^111^ | United Kingdom | 2008 | Cross-sectional | 1942 | 54.5 (11.4) | 72.7 | 22.5 | NA | 14.9 (10.6) | 60.3 | NA | 7.5 | 50.1 | ******* |
| McDonnell et al.^112^ | United Kingdom | 2001 | Cross-sectional | 248 | 49.4 (NA) | 68.9 | NA | NA | 18.5 (NA) | NA | NA | 30.0 | NA | ********* |
| Midgard et al.^113^ | Norway | 1996 | Cross-sectional | 124 | NA | 53.2 | NA | 3.8 (NA) | 7.8 (NA) | 8.9 | NA | 25.0 | NA | ******** |
| Miller et al.^114^ | Israel | 2006 | Cross-sectional | 215 | NA | 75.7 | NA | 3.0 (2.0) | 3.2 (0.8) | 31.8 | NA | 41.2 | 2.8 | ********* |
| Mitchell et al.^115^ | United Kingdom | 1981 | Cross-sectional | 197 | NA | 36.0 | NA | NA | NA | NA | 0.0 | 41.2 | 40.1 | ******** |
| Motl et al.^116^ | United States of America | 2012 | Longitudinal cohort study | 218 | 43.5 (10.0) | 90.4 | 24.0 | NA | 8.0 (6.9) | 92.0 | NA | 29.0 | NA | ******* |
| Murley et al. ^117^ | Sweden | 2020 | Longitudinal cohort study | 2553 | NA | 70.0 | 37.0 | NA | NA | NA | NA | 13.0 | NA | ********* |
| Nery-Hurwit et al.^118^ | United States of America | 2018 | Cross-sectional | 259 | 48.6 (10.5) | 84.2 | NA | NA | NA | 26.7 | NA | 42.8 | 7.7 | ******* |
| Neuberger et al.^119^ | United States of America | 2021 | Cross-sectional | 630 | NA | 62.4 | 81.6 | NA | 5.6 (5.7) | 7.0 | 100.0 | 34.4 | NA | ******** |
| Nicholas et al.^120^ | United Kingdom | 2020 | Cross-sectional | 537 | NA | NA | 70.0 | NA | NA | 40.0 | 16.0 | 41.4 | NA | ******** |
| Nickel et al.^121^ | Germany | 2018 | Cross-sectional | 1220 | NA | 76.0 | NA | NA | 11.5 (NA) | 41.0 | NA | 50.0 | NA | ******** |
| Pack et al.^122^ | United States of America | 2014 | Cross-sectional | 1310 | 50.0 (12.0) | 79.0 | 40.0 | NA | NA | NA | NA | 49.4 | NA | ******* |
| Pakenham et al.^123^ | Australia | 2008 | Cross-sectional | 232 | 48.7 (11.1) | 77.0 | 59.0 | NA | 10.4 (7.9) | 100.0 | NA | 52.0 | NA | **** |
| Patten et al.^124^ | Canada | 2012 | Cross-sectional | 245 | NA | 71.5 | 61.5 | NA | NA | NA | NA | 35.6 | NA | ********* |
| Patten et al.^125^ | Canada | 2013 | Longitudinal cohort study | 2053 | NA | 76.4 | 68.3 | NA | NA | NA | NA | 50.4 | NA | ******** |
| Patti et al.^126^ | Italy | 2007 | Cross-sectional | 593 | NA | 70.7 | 13.7 | NA | NA | NA | NA | 8.1 | NA | ********* |
| Patti et al.^127^ | Italy | 2007 | Cross-sectional | 344 | 36.9 (9.9) | 64.8 | NA | 2.5 (1.7) | 6.0 (5.4) | 16.9 | 71.8 | 62.2 | NA | ********* |
| Péntek et al.^128^ | Hungary | 2017 | Cross-sectional | 443 | 46.9 (12.0) | 78.3 | 33.6 | 3.9 (2.5) | NA | 45.0 | 57.6 | 43.8 | 7.7 | ********* |
| Pfleger et al.^129^ | Denmark | 2010 | Longitudinal cohort study | 2538 | 35.3 (NA) | 63.0 | NA | NA | 4.2 (0.2) | 95.8 | NA | 7.2 | 30.0 | ********* |
| Pluta-Fuerst et al.^130^ | Multinational | 2011 | Cross-sectional | 484 | 41.2 (9.4) | 52.3 | NA | 4.3 (2.2) | 8.6 (6.7) | 54.0 | 36.0 | 59.0 | NA | ***** |
| Ponzio et al.^131^ | Italy | 2015 | Cross-sectional | 1016 | 40.0 (9.3) | 65.2 | 26.7 | NA | 13.7 (8.6) | 86.3 | NA | 26.0 | NA | ********* |
| Ponzio et al.^132^ | Italy | 2019 | Cross-sectional | 1014 | 49.8 (12.8) | NA | 69.4 | NA | 15.3 (10.2) | NA | NA | 49.4 | NA | ********* |
| Ponzio et al.^133^ | Italy | 2020 | Cross-sectional | 1013 | 49.8 (12.8) | 69.4 | 20.4 | NA | 15.3 (10.2) | NA | 63.3 | 62.6 | NA | ******* |
| Rasmussen et al.^134^ | Denmark | 2017 | Cross-sectional | 830 | 54.3 (10.0) | 74.0 | 15.2 | 4.2 (2.4) | NA | NA | 43.3 | 41.0 | 33.7 | ********* |
| Ratajska et al.^135^ | United States of America | 2020 | Cross-sectional | 789 | 37.0 (11.2) | 75.4 | NA | 2.2 (1.3) | 13.8 (8.5) | NA | NA | 38.9 | NA | ********* |
| Riazi et al.^136^ | United Kingdom | 2003 | Cross-sectional | 638 | NA | 64 | 32.1 | NA | NA | NA | NA | 75.5 | NA | ******** |
| Roessler et al.^137^ | United States of America | 2004 | Cross-sectional | 1310 | NA | 77.9 | 40.0 | NA | NA | 32.6 | NA | 57.0 | NA | ******* |
| Roessler et al.^138^ | United States of America | 2016 | Cross-sectional | 206 | 48.7 (12.7) | 75.7 | 36.9 | NA | NA | 45.6 | NA | 56.8 | NA | ******* |
| Rozin et al.^139^ | Israel | 1982 | Interventional study | 198 | NA | 57.0 | NA | NA | NA | NA | 0.0 | 42.4 | NA | ********* |
| Rzepinski et al.^140^ | Poland | 2015 | Longitudinal cohort study | 375 | 43.1 (12.5) | 69.3 | 35.7 | 3.7 (1.7) | NA | 100.0 | 33.3 | 43.7 | NA | ****** |
| Sa et al.^141^ | Portugal | 2017 | Cross-sectional | 493 | 48.4 (11.0) | 66.5 | 38.9 | 3.8 (2.5) | NA | 46.0 | 78.9 | 46.6 | 7.9 | ********* |
| Salter et al.^142^ | United States of America | 2010 | Cross-sectional | 8180 | 53.8 (10.4) | 76.3 | 46.1 | NA | 15.5 (9.3) | 84.5 | NA | 65.9 | NA | ******** |
| Salter et al.^143^ | United States of America | 2017 | Cross-sectional | 5887 | 58.8 (10.3) | 82.0 | 44.2 | NA | 20.1 (9.9) | 19.7 | 53.6 | 50.3 | NA | ********* |
| Scheinberg et al.^144^ | Germany | 1981 | Cross-sectional | 257 | NA | 68.5 | NA | NA | NA | NA | 0.0 | 38.5 | 3.9 | ******* |
| Schmidt et al.^145^ | Germany | 2019 | Cross-sectional | 260 | 44.5 (11.2) | 79.6 | NA | 3.3 (1.9) | 10.6 (8.7) | 33.1 | 53.1 | 25.3 | 8.5 | ******* |
| Stuifbergen et al.^146^ | United States of America | 2009 | Longitudinal cohort study | 442 | 55.9 (9.7) | NA | NA | NA | NA | 55.0 | 62.0 | 34.0 | 24.0 | ******* |
| Stuifbergen et al.^147^ | United States of America | 2016 | Longitudinal cohort study | 606 | 50.6 (10.3) | 83.0 | 35.0 | NA | 13.4 (7.4) | 50.0 | NA | 32.1 | 14.5 | ******* |
| Sundstrom et al.^148^ | Sweden | 2003 | Cross-sectional | 307 | 48.8 (NA) | 42.0 | NA | 4.5 (NA) | 16.4 (NA) | 47.2 | NA | 20.2 | NA | ****** |
| Svendsen et al. ^149^ | Norway | 2018 | Cross-sectional | 546 | 53.2 (12.9) | 68.0 | 43.0 | 3.4 (2.4) | 14.0 (6.2) | NA | 40.0 | 39.0 | 11.0 | ******** |
| Taylor et al.^150^ | Australia | 2014 | Cross-sectional | 2459 | 45.0 (2.1) | 82.4 | 60.0 | NA | 7.0 (6.7) | 38.7 | NA | NA | 22.9 | ******* |
| Thompson et al.^151^ | United Kingdom | 2017 | Cross-sectional | 563 | 56.7 (10.8) | 70.1 | 38.4 | 5.5 (2.2) | NA | 49.2 | 38.7 | 55.2 | 27.5 | ********* |
| Uitdehaag et al.^152^ | Holland | 2017 | Cross-sectional | 309 | 54.0 (10.5) | 71.7 | 27.2 | 4.9 (2.3) | NA | 56.6 | 32.7 | 64.4 | 1.3 | ********* |
| van der Hiele et al.^153^ | Holland | 2021 | Cross-sectional | 241 | NA | 78.0 | 42.3 | NA | NA | NA | 77.6 | 12.9 | NA | ******* |

1. Alhussain H, Aldayel AA, Alenazi A, Alowain F. Multiple Sclerosis Patients in Saudi Arabia: Prevalence of Depression and its Extent of Severity. *Cureus*. 2020;12(2):e7005. doi:10.7759/cureus.7005

2. AlZahrani AS, Alshamrani FJ, Al-Khamis FA, et al. Association of acute stress with multiple sclerosis onset and relapse in Saudi Arabia. *Saudi Med J*. 2019;40(4):372-378. doi:10.15537/smj.2019.4.24010

3. Aronson KJ. Quality of life among persons with multiple sclerosis and their caregivers. *Neurology*. 1997;48(1):74-80. doi:10.1212/wnl.48.1.74

4. Arroyo R, Massana M, Vila C. Correlation between spasticity and quality of life in patients with multiple sclerosis: the CANDLE study. *Int J Neurosci*. 2013;123(12):850-858. doi:10.3109/00207454.2013.812084

5. Bamer AM, Cetin K, Johnson KL, Gibbons LE, Ehde DM. Validation study of prevalence and correlates of depressive symptomatology in multiple sclerosis. *Gen Hosp Psychiatry*. 2008;30(4):311-317. doi:10.1016/j.genhosppsych.2008.04.006

6. Baroin A, Chopard G, Siliman G, et al. Validation of a new quality of life scale related to multiple sclerosis and relapses. *Qual life Res an Int J Qual life Asp Treat care Rehabil*. 2013;22(8):1943-1954. doi:10.1007/s11136-012-0334-0

7. Bass AD, Van Wijmeersch B, Mayer L, et al. Effect of Multiple Sclerosis on Daily Activities, Emotional Well-being, and Relationships: The Global vsMS Survey. *Int J MS Care*. 2020;22(4):158-164. doi:10.7224/1537-2073.2018-087

8. Battaglia M, Kobelt G, Ponzio M, Berg J, Capsa D, Dalén J. New insights into the burden and costs of multiple sclerosis in Europe: Results for Italy. *Mult Scler*. 2017;23(2_suppl):104-116. doi:10.1177/1352458517708176

9. Baumstarck K, Pelletier J, Aghababian V, et al. Is the concept of quality of life relevant for multiple sclerosis patients with cognitive impairment? Preliminary results of a cross-sectional study. *PLoS One*. 2012;7(1):e30627. doi:10.1371/journal.pone.0030627

10. Becker V, Heeschen V, Schuh K, Schieb H, Ziemssen T. Patient satisfaction and healthcare services in specialized multiple sclerosis centres in Germany. *Ther Adv Neurol Disord*. 2018;11:1756285617748845. doi:10.1177/1756285617748845

11. Beier M, Hartoonian N, D’Orio VL, et al. Relationship of perceived stress and employment status in individuals with multiple sclerosis. *Work*. 2019;62(2):243-249. doi:10.3233/WOR-192859

12. Berg J, Lindgren P, Fredrikson S, Kobelt G. Costs and quality of life of multiple sclerosis in Sweden. *Eur J Heal Econ HEPAC Heal Econ Prev care*. 2006;7 Suppl 2:S75-85. doi:10.1007/s10198-006-0379-5

13. Bishop M, Frain MP, Rumrill PD, Rymond C. The relationship of self-management and disease modifying therapy use to employment status among adults with multiple sclerosis. *J Vocat Rehabil*. 2009;31(2):119-127. doi:10.3233/JVR-2009-0480

14. Bishop M, Frain MP, Espinosa CT, Stenhoff DM. Sources of information about multiple sclerosis: Information seeking and personal, demographic, and MS variables. *J Vocat Rehabil*. 2009;31(2):107-117. doi:10.3233/JVR-2009-0479

15. Bishop M, Roessler RT, Rumrill PD, et al. The relationship between housing accessibility variables and employment status among adults with multiple sclerosis. *J Rehabil*. 2013;79(4):4-14.

16. Bishop M, Chan F, Rumrill PD, et al. Employment Among Working-Age Adults With Multiple Sclerosis: A Data-Mining Approach to Identifying Employment Interventions. *Rehabil Res POLICY Educ*. 2015;29(2):135-152. doi:10.1891/2168-6653.29.2.135

17. Bishop M, Fraser R, Li J, et al. Life domains that are important to quality of life for people with multiple sclerosis: A population-based qualitative analysis. *J Vocat Rehabil*. 2019;51(1):67-76. doi:10.3233/JVR-191026

18. Bo M, Charrier L, Bartalini S, et al. Access to social security benefits among multiple sclerosis patients in Italy: A cross-sectional study. *Mult Scler Relat Disord*. 2018;24:107-112. doi:10.1016/j.msard.2018.06.016

19. Bøe Lunde HM, Telstad W, Grytten N, et al. Employment among Patients with Multiple Sclerosis-A Population Study. *PLoS One*. 2014;9(7):e103317. doi:10.1371/journal.pone.0103317

20. Boyko A, Kobelt G, Berg J, et al. New insights into the burden and costs of multiple sclerosis in Europe: Results for Russia. *Mult Scler*. 2017;23(2_suppl):155-165. doi:10.1177/1352458517708668

21. Broersma F, Oeseburg B, Dijkstra J, Wynia K. The impact of self-perceived limitations, stigma and sense of coherence on quality of life in multiple sclerosis patients: results of a cross-sectional study. *Clin Rehabil*. 2018;32(4):536-545. doi:10.1177/0269215517730670

22. Brundin L, Kobelt G, Berg J, Capsa D, Eriksson J. New insights into the burden and costs of multiple sclerosis in Europe: Results for Sweden. *Mult Scler*. 2017;23(2_suppl):179-191. doi:10.1177/1352458517708682

23. Buchanan RJ, Radin D, Chakravorty BJ, Tyry T. Informal care giving to more disabled people with multiple sclerosis. *Disabil Rehabil*. 2009;31(15):1244-1256. doi:10.1080/09638280802532779

24. Buhse M, Banker WM, Clement LM. Factors associated with health-related quality of life among older people with multiple sclerosis. *Int J MS Care*. 2014;16(1):10-19. doi:10.7224/1537-2073.2012-046

25. Calabrese P, Kobelt G, Berg J, Capsa D, Eriksson J. New insights into the burden and costs of multiple sclerosis in Europe: Results for Switzerland. *Mult Scler*. 2017;23(2_suppl):192-203. doi:10.1177/1352458517708685

26. Carnero Contentti E, Pettinicchi JP, López PA, et al. Access and unmet needs to multiple sclerosis care in a cohort of Argentinean patients. *Mult Scler Relat Disord*. 2019;33:88-93. doi:10.1016/j.msard.2019.05.024

27. Carney P, O’Boyle D, Larkin A, McGuigan C, O’Rourke K. Societal costs of multiple sclerosis in Ireland. *J Med Econ*. 2018;21(5):425-437. doi:10.1080/13696998.2018.1427100

28. Catanzaro M, Weinert C. Economic status of families living with multiple sclerosis. *Int J Rehabil Res Int Zeitschrift fur Rehabil Rev Int Rech Readapt*. 1992;15(3):209-218. doi:10.1097/00004356-199209000-00004

29. Chamot E, Kister I, Cutter GR. Item response theory-based measure of global disability in multiple sclerosis derived from the Performance Scales and related items. *BMC Neurol*. 2014;14:192. doi:10.1186/s12883-014-0192-1

30. Chen J, Taylor B V, Blizzard L, Simpson SJ, Palmer AJ, van der Mei IAF. Effects of multiple sclerosis disease-modifying therapies on employment measures using patient-reported data. *J Neurol Neurosurg Psychiatry*. 2018;89(11):1200-1207. doi:10.1136/jnnp-2018-318228

31. Chen J, Taylor B, Palmer AJ, et al. Estimating MS-related work productivity loss and factors associated with work productivity loss in a representative Australian sample of people with multiple sclerosis. *Mult Scler*. 2019;25(7):994-1004. doi:10.1177/1352458518781971

32. Chen J, Taylor B, Van der Mei I. Risk factors of leaving employment due to multiple sclerosis and changes in risk over the past decades: survival analysis with competing risks. *Mult Scler J*. 2019;25 MA-P(3):348.

33. Chiu C-Y, Fitzgerald SD, Strand DM, Muller V, Brooks J, Chan F. Motivational and Volitional Variables Associated With Stages of Change for Exercise in Multiple Sclerosis: A Multiple Discriminant Analysis. *Rehabil Couns Bull*. 2012;56(1):23-33. doi:10.1177/0034355212439898

34. Chiu CY, Tansey TN, Chan F, Strauser D, Frain MP, Arora S. Effect of Rehabilitation Technology Services on Vocational Rehabilitation Outcomes of Individuals With Multiple Sclerosis. *Rehabil Res POLICY Educ*. 2015;29(2):183-192. doi:10.1891/2168-6653.29.2.183

35. Chiu C, Park M, Hoffman T, Campbell M, Bishop M. Descriptive analysis of free-text comments on healthcare priorities and experiences in a national sample of people with multiple sclerosis. *Mult Scler Relat Disord*. 2019;34:141-149. doi:10.1016/j.msard.2019.06.023

36. Chwastiak LA, Gibbons LE, Ehde DM, et al. Fatigue and psychiatric illness in a large community sample of persons with multiple sclerosis. *J Psychosom Res*. 2005;59(5):291-298. doi:10.1016/j.jpsychores.2005.06.001

37. Clingerman E, Stuifbergen A, Becker H. The influence of resources on perceived functional limitations among women with multiple sclerosis. *J Neurosci Nurs J Am Assoc Neurosci Nurses*. 2004;36(6):312-321. doi:10.1097/01376517-200412000-00004

38. Cofield SS, Thomas N, Tyry T, Fox RJ, Salter A. Shared Decision Making and Autonomy Among US Participants with Multiple Sclerosis in the NARCOMS Registry. *Int J MS Care*. 2017;19(6):303-312. doi:10.7224/1537-2073.2016-091

39. Coleman CI, Sidovar MF, Roberts MS, Kohn C. Impact of mobility impairment on indirect costs and health-related quality of life in multiple sclerosis. *PLoS One*. 2013;8(1):e54756. doi:10.1371/journal.pone.0054756

40. da Silva NL, Takemoto MLS, Damasceno A, et al. Cost analysis of multiple sclerosis in Brazil: a cross-sectional multicenter study. *BMC Health Serv Res*. 2016;16. doi:10.1186/s12913-016-1352-3

41. D’hooghe MB, Haentjens P, Van Remoortel A, De Keyser J, Nagels G. Self-reported levels of education and disability progression in multiple sclerosis. *Acta Neurol Scand*. 2016;134(6):414-419. doi:10.1111/ane.12555

42. D’hooghe MB, De Cock A, Benedict RHB, et al. Perceived neuropsychological impairment inversely related to self-reported health and employment in multiple sclerosis. *Eur J Neurol*. 2019;26(12):1447-1454. doi:10.1111/ene.14012

43. Dubois B, Kobelt G, Berg J, Capsa D, Gannedahl M. New insights into the burden and costs of multiple sclerosis in Europe: Results for Belgium. *Mult Scler*. 2017;23(2_suppl):29-40. doi:10.1177/1352458517708100

44. Blahova Dusankova J, Kalincik T, Dolezal T, Kobelt G, Havrdova E. Cost of multiple sclerosis in the Czech Republic: the COMS study. *Mult Scler*. 2012;18(5):662-668. doi:10.1177/1352458511424422

45. Esposito S, Sparaco M, Maniscalco GT, et al. Lifestyle and Mediterranean diet adherence in a cohort of Southern Italian patients with Multiple Sclerosis. *Mult Scler Relat Disord*. 2021;47:102636. doi:https://doi.org/10.1016/j.msard.2020.102636

46. Estrutti CM, Cardoso GSME, Novais MAP de, Oliveira EML de, Bichuetti DB. Employment status of people diagnosed with multiple sclerosis in Brazil. *Arq Neuropsiquiatr*. 2019;77(5):341-345. doi:10.1590/0004-282X20190051

47. Fantoni-Quinton S, Kwiatkowski A, Vermersch P, Roux B, Hautecoeur P, Leroyer A. Impact of multiple sclerosis on employment and use of job-retention strategies: The situation in France in 2015. *J Rehabil Med*. 2016;48(6):535-540. doi:10.2340/16501977-2093

48. Farnoush R, Sahebolzamani M, Aliloo L, Rahmani A. Educational, psycho mental and socio economical needs of an iranian cohort with multiple sclerosis. *Oman Med J*. 2010;25(1):22-25. doi:10.5001/omj.2010.6

49. Fernandez O, Baumstarck-Barrau K, Simeoni M-CC, Auquier P; MusiQoL study group. Patient characteristics and determinants of quality of life in an international population with multiple sclerosis: Assessment using the MusiQoL and SF-36 questionnaires. *Mult Scler J*. 2011;17(10):1238-1249. doi:10.1177/1352458511407951

50. Fernandez-Jimenez E, Panyavin I, Perez-San-Gregorio MA, Schultheis MT. Quality of Life and Depressive Symptomatology in Multiple Sclerosis: A Cross-Sectional Study Between the USA and Spain. *Psicothema*. 2021;33(1):60-69. doi:10.7334/psicothema2020.151

51. Fidao A, De Livera A, Nag N, Neate S, Jelinek GA, Simpson-Yap S. Depression mediates the relationship between fatigue and mental health-related quality of life in multiple sclerosis. *Mult Scler Relat Disord*. 2021;47. doi:10.1016/j.msard.2020.102620

52. Flachenecker P, Kobelt G, Berg J, Capsa D, Gannedahl M. New insights into the burden and costs of multiple sclerosis in Europe: Results for Germany. *Mult Scler*. 2017;23(2_suppl):78-90. doi:10.1177/1352458517708141

53. Flensner G, Landtblom AM, Söderhamn O, Ek AC. Work capacity and health-related quality of life among individuals with multiple sclerosis reduced by fatigue: A cross-sectional study. *BMC Public Health*. 2013;13(1). doi:10.1186/1471-2458-13-224

54. Fogarty E, Walsh C, McGuigan C, Tubridy N, Barry M. Direct and indirect economic consequences of multiple sclerosis in Ireland. *Appl Health Econ Health Policy*. 2014;12(6):635-645. doi:10.1007/s40258-014-0128-3

55. Forbes A, While A, Mathes L, Griffiths P. Health problems and health-related quality of life in people with multiple sclerosis. *Clin Rehabil*. 2006;20(1):67-78. doi:10.1191/0269215506cr880oa

56. Ford HL, Gerry E, Johnson MH, Tennant A. Health status and quality of life of people with multiple sclerosis. *Disabil Rehabil*. 2001;23(12):516-521. doi:10.1080/09638280010022090

57. Garcia-Dominguez JM, Maurino J, Martinez-Gines ML, et al. Economic burden of multiple sclerosis in a population with low physical disability. *BMC Public Health*. 2019;19. doi:10.1186/s12889-019-6907-x

58. Genevie L, Kallos JE, Struening EL. Job Retention Among People with Multiple Sclerosis. *Neurorehabil Neural Repair*. 1987;1(3):131-135. doi:10.1177/136140968700100304

59. Gil-González I, Pérez-San-Gregorio MÁ, Conrad R, Martín-Rodríguez A. Predicting improvement of quality of life and mental health over 18-months in multiple sclerosis patients. *Mult Scler Relat Disord*. 2021;53:103093. doi:https://doi.org/10.1016/j.msard.2021.103093

60. Glanz BI, Dégano IR, Rintell DJ, Chitnis T, Weiner HL, Healy BC. Work productivity in relapsing multiple sclerosis: associations with disability, depression, fatigue, anxiety, cognition, and health-related quality of life. *Value Heal J Int Soc Pharmacoeconomics Outcomes Res*. 2012;15(8):1029-1035. doi:10.1016/j.jval.2012.07.010

61. Gottberg K, Einarsson U, Ytterberg C, et al. Health-related quality of life in a population-based sample of people with multiple sclerosis in Stockholm County. *Mult Scler*. 2006;12(5):605-612. doi:10.1177/1352458505070660

62. Gross HJ, Watson C. Characteristics, burden of illness, and physical functioning of patients with relapsing-remitting and secondary progressive multiple sclerosis: a cross-sectional US survey. *Neuropsychiatr Dis Treat*. 2017;13:1349-1357. doi:10.2147/NDT.S132079

63. Gulick EE, Yam M, Touw MM. Work performance by persons with multiple sclerosis: conditions that impede or enable the performance of work. *Int J Nurs Stud*. 1989;26(4):301-311. doi:10.1016/0020-7489(89)90017-5

64. Gulick EE. Reliability and validity of the work assessment scale for persons with multiple sclerosis. *Nurs Res*. 1991;40(2):107-112.

65. Gulick EE. Model for predicting work performance among persons with multiple sclerosis. *Nurs Res*. 1992;41(5):266-272.

66. Gulick EE. Health status, work impediments, and coping related to work roles of women with multiple sclerosis. *Work*. 1996;6(3):153-166. doi:10.3233/WOR-1996-6303

67. Hadjigeorgiou G, Dardiotis E, Tsivgoulis G, et al. Observational study assessing demographic, economic and clinical factors associated with access and utilization of health care services of patients with multiple sclerosis under treatment with interferon beta-1b (EXTAVIA). *PLoS One*. 2014;9(11):e113933. doi:10.1371/journal.pone.0113933

68. Hategeka C, Traboulsee AL, McMullen K, Lynd LD. Association of Unemployment and Informal Care with Stigma in Multiple Sclerosis: Evidence from the Survey on Living with Neurological Conditions in Canada. *Int J MS Care*. 2019;21(5):214-225. doi:10.7224/1537-2073.2017-108

69. Heinzlef O, Molinier G, van Hille B, Radoszycki L, Dourgnon P, Longin J. Economic Burden of the Out-of-Pocket Expenses for People with Multiple Sclerosis in France. *PharmacoEconomics - open*. 2020;4(4):593-603. doi:10.1007/s41669-020-00199-7

70. Henriksson F, Fredrikson S, Masterman T, Jönsson B. Costs, quality of life and disease severity in multiple sclerosis: a cross-sectional study in Sweden. *Eur J Neurol*. 2001;8(1):27-35. doi:10.1046/j.1468-1331.2001.00169.x

71. Honan CA, Brown RF, Hine DW. The multiple sclerosis work difficulties questionnaire (MSWDQ): development of a shortened scale. *Disabil Rehabil*. 2014;36(8):635-641. doi:10.3109/09638288.2013.805258

72. Iezzoni LI, Ngo L. Health, disability, and life insurance experiences of working-age persons with multiple sclerosis. *Mult Scler*. 2007;13(4):534-546. doi:10.1177/1352458506071356

73. Iezzoni LI, Rao SR, Kinkel RP. Experiences acquiring and using mobility aids among working-age persons with multiple sclerosis living in communities in the United States. *Am J Phys Med Rehabil*. 2010;89(12):1010-1023. doi:10.1097/PHM.0b013e3181f70292

74. Imani A, Gharibi F, Khezri A, Joudyian N, Dalal K. Economic costs incurred by the patients with multiple sclerosis at different levels of the disease: a cross-sectional study in Northwest Iran. *BMC Neurol*. 2020;20(1):205. doi:10.1186/s12883-020-01790-5

75. Iwanaga K, Wu J-R, Chen X, et al. Person-environment contextual factors as mediators for the relationship between symptom cluster and employment outcome in multiple sclerosis. *J Vocat Rehabil*. 2018;48(2):197-206. doi:10.3233/JVR-180930

76. Jennum P, Wanscher B, Frederiksen J, Kjellberg J. The socioeconomic consequences of multiple sclerosis: a controlled national study. *Eur Neuropsychopharmacol J Eur Coll Neuropsychopharmacol*. 2012;22(1):36-43. doi:10.1016/j.euroneuro.2011.05.001

77. Johansson S, Ytterberg C, Gottberg K, Holmqvist LW, von Koch L, Conradsson D. Participation in social/lifestyle activities in people with multiple sclerosis: Changes across 10 years and predictors of sustained participation. *Mult Scler*. 2020;26(13):1775-1784. doi:10.1177/1352458519881991

78. Johansson S, Skjerbæk AG, Nørgaard M, Boesen F, Hvid LG, Dalgas U. Associations between fatigue impact and lifestyle factors in people with multiple sclerosis - The Danish MS hospitals rehabilitation study. *Mult Scler Relat Disord*. 2021;50:102799. doi:10.1016/j.msard.2021.102799

79. Johnson KL, Bamer AM, Fraser RT. Disease and demographic characteristics associated with unemployment among working-age adults with multiple sclerosis. *Int J MS Care*. 2013;15(SUPPL.1):44-51. doi:10.7224/1537-2073-15.S1.44

80. Jones E, Pike J, Marshall T, Ye X. Quantifying the relationship between increased disability and health care resource utilization, quality of life, work productivity, health care costs in patients with multiple sclerosis in the US. *BMC Health Serv Res*. 2016;16:294. doi:10.1186/s12913-016-1532-1

81. Julian LJ, Vella L, Vollmer T, Hadjimichael O, Mohr DC. Employment in multiple sclerosis. Exiting and re-entering the work force. *J Neurol*. 2008;255(9):1354-1360. doi:10.1007/s00415-008-0910-y

82. Kalantari S, Karbakhsh M, Kamiab Z, Kalantari Z, Sahraian MA. Perceived Social Stigma in Patients with Multiple Sclerosis: A Study from Iran. *Acta Neurol Taiwan*. 2018;27(1):1-8.

83. Kallmann BA, Tiel-Wilck K, Kullmann JS, Engelmann U, Chan A. Real-life outcomes of teriflunomide treatment in patients with relapsing multiple sclerosis: TAURUS-MS observational study. *Ther Adv Neurol Disord*. 2019;12:1756286419835077. doi:10.1177/1756286419835077

84. Khader H Al, Emran B, Sulaimi M Al, et al. Estimating the prevalence of cognition and mental health among multiple sclerosis patients: A population-based cross-sectional study. *Mult Scler Relat Disord*. 2019;36:101391. doi:https://doi.org/10.1016/j.msard.2019.101391

85. Kirk-Brown AK, Van Dijk PA, Simmons RD, Bourne MP, Cooper BK. Disclosure of diagnosis of multiple sclerosis in the workplace positively affects employment status and job tenure. *Mult Scler*. 2014;20(7):871-876. doi:10.1177/1352458513513967

86. Kobelt G, Lindgren P, Smala A, et al. Costs and quality of life in multiple sclerosis. An observational study in Germany. *HEPAC Heal Econ Prev Care*. 2001;2(2):60-68.

87. Kobelt G, Berg J, Lindgren P, et al. Costs and quality of life of multiple sclerosis in Austria. *Eur J Heal Econ HEPAC Heal Econ Prev care*. 2006;7 Suppl 2:S14-23. doi:10.1007/s10198-006-0382-x

88. Kobelt G. Costs and quality of life for patients with multiple sclerosis in Belgium. *Eur J Heal Econ HEPAC Heal Econ Prev care*. 2006;7 Suppl 2:S24-33. doi:10.1007/s10198-006-0377-7

89. Kobelt G, Berg J, Lindgren P, Kerrigan J, Russell N, Nixon R. Costs and quality of life of multiple sclerosis in the United Kingdom. *Eur J Heal Econ HEPAC Heal Econ Prev care*. 2006;7 Suppl 2:S96-104. doi:10.1007/s10198-006-0380-z

90. Kobelt G, Berg J, Lindgren P, et al. Costs and quality of life in multiple sclerosis in The Netherlands. *Eur J Heal Econ HEPAC Heal Econ Prev care*. 2006;7 Suppl 2:S55-64. doi:10.1007/s10198-006-0378-6

91. Kobelt G, Berg J, Lindgren P, Battaglia M, Lucioni C, Uccelli A. Costs and quality of life of multiple sclerosis in Italy. *Eur J Heal Econ HEPAC Heal Econ Prev care*. 2006;7 Suppl 2:S45-54. doi:10.1007/s10198-006-0385-7

92. Kobelt G, Berg J, Lindgren P, Izquierdo G, Sánchez-Soliño O, Pérez-Miranda J, et al. Costs and quality of life of multiple sclerosis in Spain. *Eur J Heal Econ HEPAC Heal Econ Prev care*. 2006;7 Suppl 2:S65-74. doi:10.1007/s10198-006-0381-y

93. Kobelt G, Berg J, Lindgren P, Gerfin A, Lutz J. Costs and quality of life of multiple sclerosis in Switzerland. *Eur J Heal Econ HEPAC Heal Econ Prev care*. 2006;7 Suppl 2:S86-95. doi:10.1007/s10198-006-0383-9

94. Kobelt G, Berg J, Lindgren P, et al. Costs and quality of life of multiple sclerosis in Germany. *Eur J Heal Econ HEPAC Heal Econ Prev care*. 2006;7 Suppl 2:S34-44. doi:10.1007/s10198-006-0384-8

95. Kobelt G, Berg J, Atherly D, Hadjimichael O. Costs and quality of life in multiple sclerosis: a cross-sectional study in the United States. *Neurology*. 2006;66(11):1696-1702. doi:10.1212/01.wnl.0000218309.01322.5c

96. Selmaj K, Kobelt G, Berg J, Orlewska E, Capsa D, Dalén J. New insights into the burden and costs of multiple sclerosis in Europe: Results for Poland. *Mult Scler*. 2017;23(2_suppl):130-142. doi:10.1177/1352458517708666

97. Kobelt G, Teich V, Cavalcanti M, Canzonieri AM. Burden and cost of multiple sclerosis in Brazil. *PLoS One*. 2019;14(1):e0208837. doi:10.1371/journal.pone.0208837

98. Koch LC, Rumrill Jr. PD, Roessler RT, Fitzgerald S. Illness and demographic correlates of quality of life among people with multiple sclerosis. *Rehabil Psychol*. 2001;46(2):154-164. doi:10.1037/0090-5550.46.2.154

99. Kohn CG, Coleman CI, White CM, Sidovar MF, Sobieraj DM. Mobility, walking and physical activity in persons with multiple sclerosis. *Curr Med Res Opin*. 2014;30(9):1857-1862. doi:10.1185/03007995.2014.921147

100. Kornblith AB, La Rocca NG, Baum HM. Employment in individuals with multiple sclerosis. *Int J Rehabil Res Int Zeitschrift fur Rehabil Rev Int Rech Readapt*. 1986;9(2):155-165. doi:10.1097/00004356-198606000-00006

101. Kos D, Ferdinand S, Duportail M, et al. Assessing life balance of European people with multiple sclerosis: A multicenter clinimetric study within the RIMS network. *Mult Scler Relat Disord*. 2020;39:101879. doi:https://doi.org/10.1016/j.msard.2019.101879

102. Krause JS, Dismuke-Greer CE, Jarnecke M, Li C, Reed KS, Rumrill P. Employment and Gainful Earnings Among Those With Multiple Sclerosis. *Arch Phys Med Rehabil*. 2019;100(5):931-937.e1. doi:10.1016/j.apmr.2018.11.005

103. Krause JS, Li C, Backus D, et al. Barriers and Facilitators to Employment: A Comparison of Participants With Multiple Sclerosis and Spinal Cord Injury. *Arch Phys Med Rehabil*. Published online March 2021. doi:10.1016/j.apmr.2021.02.015

104. Krokavcova M, Nagyova I, van Dijk JP, et al. Mastery, functional disability and perceived health status in patients with multiple sclerosis. *Eur J Neurol*. 2008;15(11):1237-1244. doi:10.1111/j.1468-1331.2008.02304.x

105. Larocca N, Kalb R, Scheinberg L, Kendall P. Factors associated with unemployment of patients with multiple sclerosis. *J Chronic Dis*. 1985;38(2):203-210. doi:10.1016/0021-9681(85)90093-1

106. Lebrun-Frenay C, Kobelt G, Berg J, Capsa D, Gannedahl M. New insights into the burden and costs of multiple sclerosis in Europe: Results for France. *Mult Scler*. 2017;23(2_suppl):65-77. doi:10.1177/1352458517708125

107. Lehmann AI, Rodgers S, Kamm CP, et al. Factors associated with employment and expected work retention among persons with multiple sclerosis: findings of a cross-sectional citizen science study. *J Neurol*. 2020;267(10):3069-3082. doi:10.1007/s00415-020-09973-3

108. Li J, Fitzgerald SM, Bishop M, Rumrill PD, Wang F. Disease-related and functional predictors of employment status among adults with multiple sclerosis. *Work*. 2015;52(4):789-797. doi:10.3233/WOR-152206

109. Marck CH, De Livera AM, Brown CR, et al. Health outcomes and adherence to a healthy lifestyle after a multimodal intervention in people with multiple sclerosis: Three year follow-up. *PLoS One*. 2018;13(5). doi:10.1371/journal.pone.0197759

110. Maurino J, Martínez-Ginés ML, García-Domínguez JM, et al. Workplace difficulties, health-related quality of life, and perception of stigma from the perspective of patients with Multiple Sclerosis. *Mult Scler Relat Disord*. 2020;41:102046. doi:10.1016/j.msard.2020.102046

111. McCrone P, Heslin M, Knapp M, Bull P, Thompson A. Multiple sclerosis in the UK: service use, costs, quality of life and disability. *Pharmacoeconomics*. 2008;26(10):847-860. doi:10.2165/00019053-200826100-00005

112. McDonnell G V, Hawkins SA. An assessment of the spectrum of disability and handicap in multiple sclerosis: a population-based study. *Mult Scler*. 2001;7(2):111-117. doi:10.1177/135245850100700207

113. Midgard R, Riise T, Nyland H. Impairment, disability and handicap in multiple sclerosis - A cross-sectional study in an incident cohort in More and Romsdal County, Norway. *J Neurol*. 1996;243(4):337-344. doi:10.1007/BF00868408

114. Miller A, Dishon S. Health-related quality of life in multiple sclerosis: The impact of disability, gender and employment status. *Qual life Res an Int J Qual life Asp Treat care Rehabil*. 2006;15(2):259-271. doi:10.1007/s11136-005-0891-6

115. Mitchell JN. Multiple Sclerosis and the Prospects for Employment. *J Soc Occup Med*. 1981;31(4):134-138. doi:10.1093/occmed/31.4.134

116. Motl RW, Suh Y, Weikert M, Dlugonski D, Balantrapu S, Sandroff B. Fatigue, depression, and physical activity in relapsing-remitting multiple sclerosis: Results from a prospective, 18-month study. *Mult Scler Relat Disord*. 2012;1(1):43-48. doi:https://doi.org/10.1016/j.msard.2011.08.003

117. Murley C, Karampampa K, Alexanderson K, Hillert J, Friberg E. Diagnosis-specific sickness absence and disability pension before and after multiple sclerosis diagnosis: An 8-year nationwide longitudinal cohort study with matched references. *Mult Scler Relat Disord*. 2020;42:102077. doi:10.1016/j.msard.2020.102077

118. Nery-Hurwit M, Yun J, Ebbeck V. Examining the roles of self-compassion and resilience on health-related quality of life for individuals with Multiple Sclerosis. *Disabil Health J*. 2018;11(2):256-261. doi:https://doi.org/10.1016/j.dhjo.2017.10.010

119. Neuberger EE, Abbass IM, Jones E, Engmann NJ. Work Productivity Outcomes Associated with Ocrelizumab Compared with Other Disease-Modifying Therapies for Multiple Sclerosis. *Neurol Ther*. 2021;10(1):183-196. doi:10.1007/s40120-020-00224-1

120. Nicholas RS, Heaven ML, Middleton RM, et al. Personal and societal costs of multiple sclerosis in the UK: A population-based MS Registry study. *Mult Scler J - Exp Transl Clin*. 2020;6(1):2055217320901727. doi:10.1177/2055217320901727

121. Nickel S, von dem Knesebeck O, Kofahl C. Self-assessments and determinants of HRQoL in a German MS population. *Acta Neurol Scand*. 2018;137(2):174-180. doi:10.1111/ane.12854

122. Pack TG, Szirony GM, Kushner JD, Bellaw JR. Quality of life and employment in persons with multiple sclerosis. *Work*. 2014;49(2):281-287. doi:10.3233/WOR-131711

123. Pakenham KI. The nature of sense making in caregiving for persons with multiple sclerosis. *Disabil Rehabil*. 2008;30(17):1263-1273. doi:10.1080/09638280701610320

124. Patten SB, Williams JVA, Lavorato DH, Berzins S, Metz LM, Bulloch AGM. Health status, stress and life satisfaction in a community population with MS. *Can J Neurol Sci*. 2012;39(2):206-212. doi:10.1017/S031716710001324X

125. Patten SB, Williams JVA, Lavorato DH, Koch M, Metz LM. Depression as a predictor of occupational transition in a multiple sclerosis cohort. *Funct Neurol*. 2013;28(4):275-280. doi:10.11138/FNeur/2013.28.4.275

126. Patti F, Pozzilli C, Montanari E, et al. Effects of education level and employment status on HRQoL in early relapsing-remitting multiple sclerosis. *Mult Scler*. 2007;13(6):783-791. doi:10.1177/1352458506073511

127. Patti F, Russo P, Pappalardo A, Macchia F, Civalleri L, Paolillo A. Predictors of quality of life among patients with multiple sclerosis: An Italian cross-sectional study. *J Neurol Sci*. 2007;252(2):121-129. doi:https://doi.org/10.1016/j.jns.2006.10.017

128. Péntek M, Kobelt G, Berg J, et al. New insights into the burden and costs of multiple sclerosis in Europe: Results for Hungary. *Mult Scler*. 2017;23(2_suppl):91-103. doi:10.1177/1352458517708142

129. Pfleger CCH, Flachs EM, Koch-Henriksen N, Hilt Pfleger CC, Meulengracht Flachs E, Koch-Henriksen N. Social consequences of multiple sclerosis (1): Early pension and temporary unemployment-a historical prospective cohort study. *Mult Scler*. 2010;16(1):121-126. doi:10.1177/1352458509352196

130. Pluta-Fuerst A, Petrovic K, Berger T, et al. Patient-reported quality of life in multiple sclerosis differs between cultures and countries: a cross-sectional Austrian-German-Polish study. *Mult Scler*. 2011;17(4):478-486. doi:10.1177/1352458510391341

131. Ponzio M, Brichetto G, Zaratin P, Battaglia MA. Workers with disability: the case of multiple sclerosis. *Neurol Sci.* 2015;36(10):1835-1841. doi:10.1007/s10072-015-2265-3

132. Ponzio M, Tacchino A, Vaccaro C, Brichetto G, Battaglia MA, Messmer Uccelli M. Disparity between perceived needs and service provision: a cross-sectional study of Italians with multiple sclerosis. *Neurol Sci.* 2019;40(6):1137-1144. doi:10.1007/s10072-019-03780-z

133. Ponzio M, Tacchino A, Vaccaro C, et al. Unmet needs influence health-related quality of life in people with multiple sclerosis. *Mult Scler Relat Disord*. 2020;38:101877. doi:https://doi.org/10.1016/j.msard.2019.101877

134. Rasmussen PV, Kobelt G, Berg J, Capsa D, Gannedahl M. New insights into the burden and costs of multiple sclerosis in Europe: Results for Denmark. *Mult Scler*. 2017;23(2_suppl):53-64. doi:10.1177/1352458517708118

135. Ratajska A, Glanz BI, Chitnis T, Weiner HL, Healy BC. Social support in multiple sclerosis: Associations with quality of life, depression, and anxiety. *J Psychosom Res*. 2020;138:110252. doi:https://doi.org/10.1016/j.jpsychores.2020.110252

136. Riazi A, Hobart JC, Fitzpatrick R, Freeman JA, Thompson AJ. Socio-demographic variables are limited predictors of health status in multiple sclerosis. *J Neurol*. 2003;250(9):1088-1093. doi:10.1007/s00415-003-0160-y

137. Roessler RT, Rumrill PD, Fitzgerald SM. Predictors of Employment Status for People with Multiple Sclerosis. *Rehabil Couns Bull*. 2004;47(2):96-103. doi:10.1177/00343552030470020401

138. Roessler RT, Rumrill PD, Li J, Daly K, Anhalt K. High-priority employment concerns of Hispanics/Latinos with multiple sclerosis in the United States. *J Vocat Rehabil*. 2016;45(2):121-131. doi:10.3233/JVR-160817

139. Rozin R, Schiff Y, Cooper G, Kahana E. Vocational rehabilitation of multiple sclerosis (MS) patients I. Analysis of clinical and demographic factors first in a series of articles. *Disabil Rehabil*. 1982;4(2):75-79. doi:10.3109/09638288209166882

140. Rzepinski L, Zawadka-Kunikowska M, Kucharczuk J, Newton J, Zalewski P. New insights into the socio-economic aspects of multiple sclerosis in a cohort of Polish patients. *Ann Agric Environ Med*. 2021;28(1):99-106. doi:10.26444/aaem/117962

141. Sa MJ, Kobelt G, Berg J, Capsa D, Dalen J; European Multiple Sclerosis Platform. New insights into the burden and costs of multiple sclerosis in Europe: Results for Portugal. *Mult Scler J*. 2017;23:143-154. doi:10.1177/1352458517708667

142. Salter AR, Cutter GR, Tyry T, Marrie RA, Vollmer T. Impact of loss of mobility on instrumental activities of daily living and socioeconomic status in patients with MS. *Curr Med Res Opin*. 2010;26(2):493-500. doi:10.1185/03007990903500649

143. Salter A, Thomas N, Tyry T, Cutter G, Marrie RA. Employment and absenteeism in working-age persons with multiple sclerosis. *J Med Econ*. 2017;20(5):493-502. doi:10.1080/13696998.2016.1277229

144. Scheinberg L, Holland N, Larocca N, Laitin P, Bennett A, Hall H. Vocational disability and rehabilitation in multiple sclerosis. *Int J Rehabil Res Int.* 1981;4(1):61-64. doi:10.1097/00004356-198103000-00008

145. Schmidt S, Jöstingmeyer P. Depression, fatigue and disability are independently associated with quality of life in patients with multiple Sclerosis: Results of a cross-sectional study. *Mult Scler Relat Disord*. 2019;35:262-269. doi:10.1016/j.msard.2019.07.029

146. Stuifbergen A, Brown A, Phillips L. Predictors and moderators of the disablement process in persons with multiple sclerosis. *NeuroRehabilitation*. 2009;24(2):119-129. doi:10.3233/NRE-2009-0461

147. Stuifbergen AK, Blozis S, Becker H, Harrison T, Kullberg V. Selected health behaviors moderate the progression of functional limitations in persons with multiple sclerosis: Eleven years of annual follow-up. *Disabil Health J*. 2016;9(3):472-478. doi:10.1016/j.dhjo.2016.01.006

148. Sundström P, Nyström L, Svenningsson A, Forsgren L. Sick leave and professional assistance for multiple sclerosis individuals in Vasterbotten County, northern Sweden. *Mult Scler*. 2003;9(5):515-520. doi:10.1191/1352458503ms955oa

149. Svendsen B, Grytten N, Bø L, Aarseth H, Smedal T, Myhr K-M. The economic impact of multiple sclerosis to the patients and their families in Norway. *Eur J Heal Econ.* 2018;19(9):1243-1257. doi:10.1007/s10198-018-0971-5

150. Taylor KL, Hadgkiss EJ, Jelinek GA, et al. Lifestyle factors, demographics and medications associated with depression risk in an international sample of people with multiple sclerosis. *BMC Psychiatry*. 2014;14:327. doi:10.1186/s12888-014-0327-3

151. Thompson A, Kobelt G, Berg J, Capsa D, Eriksson J, Miller D. New insights into the burden and costs of multiple sclerosis in Europe: Results for the United Kingdom. *Mult Scler*. 2017;23(2_suppl):204-216. doi:10.1177/1352458517708687

152. Uitdehaag B, Kobelt G, Berg J, Capsa D, Dalén J. New insights into the burden and costs of multiple sclerosis in Europe: Results for the Netherlands. *Mult Scler*. 2017;23(2_suppl):117-129. doi:10.1177/1352458517708663

153. van der Hiele K, van Gorp DAMAM, van Egmond EEAEA, et al. Self-reported occupational functioning in persons with relapsing-remitting multiple sclerosis: Does personality matter? *J Neurol Sci*. 2021;427:117561. doi:10.1016/j.jns.2021.117561
