# Supplementary figures and images for: Spatial and temporal distribution of the prevalence of unemployment and early retirement in people with multiple sclerosis: A systematic review with meta-analysis
Source: PLoS One. 2022 Jul 28;17(7):e0272156. doi: 10.1371/journal.pone.0272156 (PMC9333213; doi:10.1371/journal.pone.0272156)

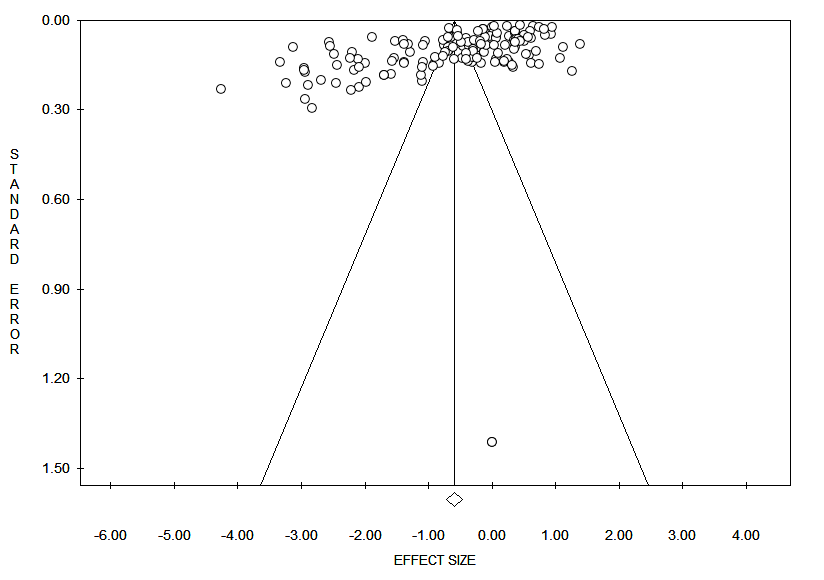

Supplement: S1 Fig — (DOCX) [file pone.0272156.s004.docx]

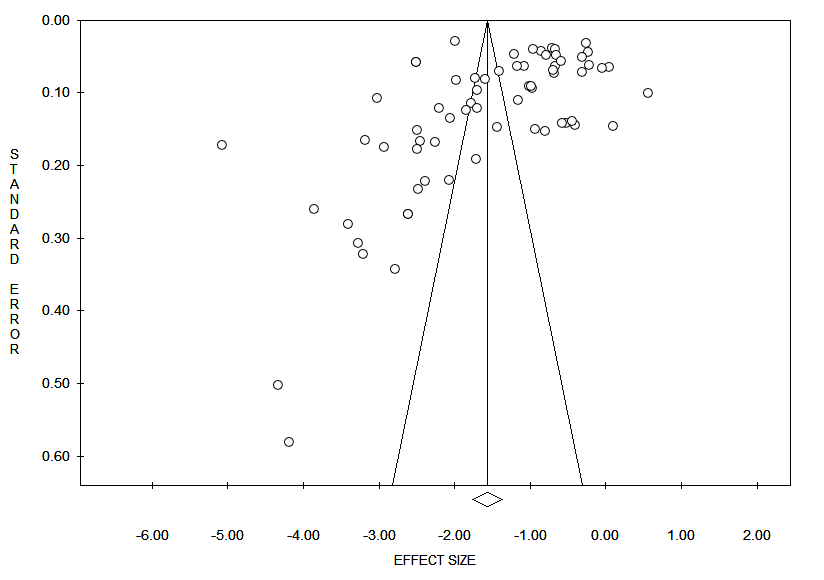

Supplement: S2 Fig — (DOCX) [file pone.0272156.s005.docx]

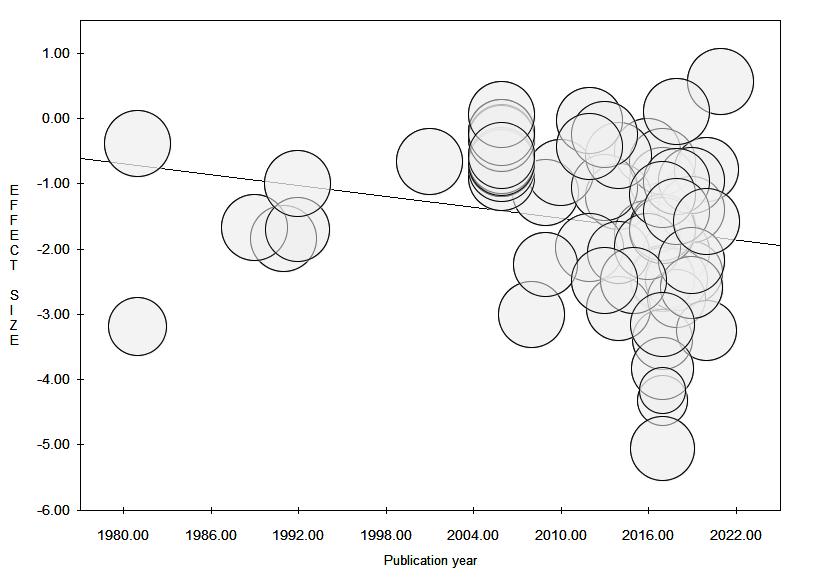

Supplement: S3 Fig — (DOCX) [file pone.0272156.s006.docx]
